# Spatio-chromatic contrast sensitivity under mesopic and photopic light levels

**DOI:** 10.1167/jov.20.4.23

**Published:** 2020-04-29

**Authors:** Sophie Wuerger, Maliha Ashraf, Minjung Kim, Jasna Martinovic, María Pérez-Ortiz, Rafał K. Mantiuk

**Affiliations:** Department of Psychology, University of Liverpool, Liverpool, UK; Department of Psychology, University of Liverpool, Liverpool, UK; Department of Computer Science and Technology, University of Cambridge, Cambridge, UK; School of Psychology, University of Aberdeen Kings College, Aberdeen, UK; Department of Computer Science, University College London, London, UK; Department of Computer Science and Technology, University of Cambridge, Cambridge, UK

**Keywords:** contrast sensitivity functions, color vision, luminance, high light level, mesopic, photopic, isoluminance, spatial vision, chromatic, achromatic, cone adaptation, light adaptation, HDR

## Abstract

Contrast sensitivity functions (CSFs) characterize the sensitivity of the human visual system at different spatial scales, but little is known as to how contrast sensitivity for achromatic and chromatic stimuli changes from a mesopic to a highly photopic range reflecting outdoor illumination levels. The purpose of our study was to further characterize the CSF by measuring both achromatic and chromatic sensitivities for background luminance levels from 0.02 cd/m^2^ to 7,000 cd/m^2^. Stimuli consisted of Gabor patches of different spatial frequencies and angular sizes, varying from 0.125 to 6 cpd, which were displayed on a custom high dynamic range (HDR) display with luminance levels up to 15,000 cd/m^2^. Contrast sensitivity was measured in three directions in color space, an achromatic direction, an isoluminant “red-green” direction, and an S-cone isolating “yellow-violet” direction, selected to isolate the luminance, L/M-cone opponent, and S-cone opponent pathways, respectively, of the early postreceptoral processing stages. Within each session, observers were fully adapted to the fixed background luminance (0.02, 2, 20, 200, 2,000, or 7,000 cd/m^2^). Our main finding is that the background luminance has a differential effect on achromatic contrast sensitivity compared to chromatic contrast sensitivity. The achromatic contrast sensitivity increases with higher background luminance up to 200 cd/m^2^ and then shows a sharp decline when background luminance is increased further. In contrast, the chromatic sensitivity curves do not show a significant sensitivity drop at higher luminance levels. We present a computational luminance-dependent model that predicts the CSF for achromatic and chromatic stimuli of arbitrary size.

## Introduction

Spatial vision refers to the ability to see image intensity variations across space. Early measurements of spatial visual sensitivity have focused on spatial resolution and spatial acuity (e.g., Shlaer, 1937) and summation of signals across space (Ricco's law; Graham & Margaria, 1935). Campbell and Robson (1968) were the first to use principles of Fourier analysis to study spatial sensitivity and introduced the contrast sensitivity function, which is the reciprocal of the threshold contrast over a range of spatial frequencies.

Since the seminal article by Campbell and Robson (1968), progress has been made in our understanding of how spatial sensitivity varies with eccentricity (Robson & Graham, 1981), pattern size (Rovamo et al., 1993; Noorlander et al., 1980), spatial orientation (Campbell et al., 1966), and mean luminance level (Mustonen et al., 1993; Van Nes & Bouman, 1967). The majority of these studies have focused on contrast sensitivity for achromatic image variations, and a comprehensive model for achromatic spatial detection mechanisms has been proposed by Watson and Ahumada (2005).

The contrast sensitivity function for chromatic modulations has been studied to a lesser degree, with some notable exceptions (Green, 1968; Cropper, 1998; Andrews & Pollen, 1979; Granger & Heurtley, 1973; van der Horst & Bouman, 1969; Kim et al., 2017; McKeefry et al., 2001; Swanson, 1996; Valero et al., 2004; Lucassen et al., 2018). The most extensive set of chromatic contrast sensitivity measurements comes from Mullen (1985) and Anderson et al. (1991), who have assessed the contrast sensitivity for isoluminant red-green and S-cone isolating (lime-violet) gratings with individually adjusted isoluminance points to isolate chromatic channels and silence the luminance-driven mechanisms. Sekiguchi et al. (1993) employed interference fringes to measure chromatic and luminance contrast sensitivity, thereby eliminating optical blur in addition to chromatic aberration; their contrast sensitivity data are in agreement with the measurements by Anderson et al. (1991).

With the advent of high-dynamic range displays, it is vital to understand how the visual system operates at very high and very low luminance levels. For achromatic contrast modulations, Van Nes and Bouman (1967) and Mustonen et al. (1993) characterized the dependence of the contrast sensitivity on light levels up to 5,900 trolands (Van Nes & Bouman, 1967). There are no corresponding measurements for chromatic contrast sensitivity. The purpose of our study is to provide a comprehensive set of measurements and a computational model of contrast sensitivity for achromatic and chromatic modulations as a function of light level, reflecting the contrast sensitivity of an average (standard) observer. Contrast sensitivity function (CSF) models reflecting the visual system of a standard observer afford the generality necessary for practical applications.

Due to the aforementioned purpose, the current study approaches the characterization of chromatic contrast sensitivity slightly differently from Mullen (1985). Truly isoluminant stimuli are difficult to achieve even when using a heterochromatic flicker paradigm (Wagner & Boynton, 1972). There are many possible sources of luminance intrusion, including interobserver variations in *V*(λ) (Gibson & Tyndall, 1923), retinal illuminance (Ikeda & Shimozono, 1981), chromatic aberration (Flitcroft, 1989), and the variation of the isoluminance point across the visual field (Bilodeau & Faubert, 1997). Therefore, rather than experimentally controlling for luminance intrusion, we instead allowed for the possibility that the stimuli are not perfectly isoluminant for each observer and included luminance intrusion in our model of chromatic channels. Since our aim is to provide a model of chromatic contrast sensitivity for an average (standard) observer that would be applicable to complex spatio-chromatic images (e.g., To & Tolhurst, 2019), it is not useful to optimize stimulus parameters for a small set of individual observers.

In the main experiment (Experiment 1), we measured contrast thresholds for three directions in color space: Stimuli were either modulated along an achromatic direction, a red-green direction (RG), or an S-cone-isolating, lime-violet direction (YV). Thresholds were measured as a function of spatial frequency (0.5, 1, 2, 4, 6 cpd) under steady-state adaptation to low mesopic (0.02 cd/m^2^) and high photopic (7,000 cd/m^2^) light levels. The subsequent experiments served as controls or were necessary to formulate a more general model. In Experiment 2, we tested whether the contrast sensitivity at medium to high luminance levels could be affected by incomplete adaptation, by measuring the contrast sensitivity with the room light on and bright diffuse lights near the stimuli. In Experiment 3, we measured the contrast sensitivity for two additional lower spatial frequencies (0.125 cpd, 0.25 cpd) to evaluate whether the chromatic contrast sensitivity has indeed a low-pass shape (Mullen, 1985) or whether, at sufficiently low spatial frequencies, the contrast sensitivity drops as it does for achromatic modulations. In Experiment 4, additional contrast sensitivity data were collected for two more envelope sizes for each spatial frequency to assess spatial summation for the three contrast modulations, which will allow us to generalize our model predictions from the fixed-cycle stimuli to arbitrary stimuli. In Experiment 1, we standardized the width of the Gaussian envelope to the spatial frequency of the underlying sine wave, so that we can treat the width of the Gaussian as a fixed parameter. This is useful for modeling, since we can then treat the width of the Gaussian as a free parameter for predicting contrast sensitivity to stimuli of different sizes.

## Experiment 1: Light level and spatial frequency

In Experiment 1, we tested how contrast sensitivity to both achromatic and chromatic contrast modulations is dependent on the background light level. We measured contrast thresholds for Gabor patches at mean luminances ranging from 0.02  cd/m^2^ (low mesopic range) to 7,000  cd/m^2^ (high photopic range).

### Methods

#### Observers

We recruited five observers from the University of Cambridge and 16 observers from the University of Liverpool. Observers provided informed consent prior to participation, in accordance with the ethical approval of respective University Ethics Committees. All naive observers were reimbursed for their time.

Eleven of the observers were naive to the purpose of the study (five female, 11 male, mean age = 26.8 ± 7.7); the rest were the authors (four female, one male, mean age = 40.4 ± 12.6). All observers had normal or corrected-to-normal visual acuity. All observers had normal color vision, verified using the Cambridge Color Test for the CRS ViSaGe System (Mollon & Reffin, 1989) or Ishihara's Tests for Colour Deficiency, 38-plates edition.

In order to verify that the experimental setups in the two locations were calibrated to the same standard, three observers repeated the experiment in both Cambridge and Liverpool. We found that the data from these observers were consistent across location and report only pooled data from these observers.

#### Apparatus

**Figure 1.**
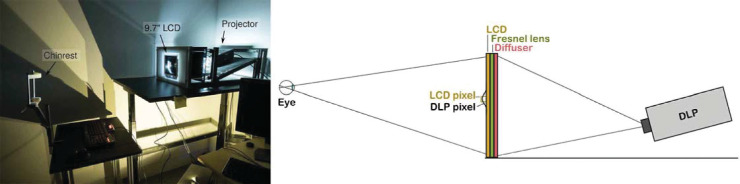
Left: a photograph of the HDR display in Cambridge. Right: the schematic diagram of the HDR display design. The image from the DLP is projected on a diffuser and further modulated by an LCD panel with its backlight removed. To improve the light efficiency of the system, a Fresnel lens with a focal length of 32 cm was introduced next to the diffuser such that the light was directed toward the eyes of the observer.

The stimuli were displayed on two custom-built high-dynamic-range (HDR) displays: one in Liverpool (peak luminance: 4,000 cd/m^2^) and one in Cambridge (peak luminance: 15,000 cd/m^2^). As the two displays were otherwise identical in construction, we describe the display in Cambridge and flag the differences. The HDR display consisted of an LCD panel (9.7 in., 2,048-pixel × 1,536-pixel iPad 3/4 retina display; product code: LG LP097QX1) and a DLP (Digital Light Processing) projector (Optoma X600 in Cambridge, Acer P1276 in Liverpool; both 1,024 × 768 pixels). The backlight of the LCD was removed and the DLP acted as the replacement backlight (Seetzen et al., 2004); see the schematic diagram (Figure 1). Because we could modulate both the pixels on the LCD and on the DLP, the maximum contrast we could achieve was a product of the contrast of each display; given 1,000:1 contrast of the LCD and 1,000:1 contrast of the DLP, the maximum contrast of our display was 1,000,000:1. The image on such a display is formed by factorizing the target image, in a linear color space, into the DLP and LCD components, such that their product forms the desired image. The factorization was performed using the original method from Seetzen et al. (2004).

Several steps were taken to improve the light efficiency and therefore the brightness of the display. The DLP had its color wheel removed, increasing its brightness by a factor of 3. The color wheel was unnecessary as the LCD panel was responsible for forming a color image. A Fresnel lens with the focal length of 32 cm was introduced behind the LCD panel to ensure that most of the light was directed toward the observer.

The display was calibrated and driven by custom-made software, written in MATLAB and relying on Psychtoolbox and MATLAB OpenGL extensions (Kleiner et al., 2007). The calibration involved displaying a series of grids consisting of dots, individually on the LCD and DLP; photographing them with a DSLR camera (Canon 550D); and finding both homographic and mesh-based transformations between DLP and LCD pixel coordinates. This step ensured an accurate alignment between LCD and DLP pixels. To compensate for spatial nonuniformity, a photograph of the display showing a uniform field was taken and used to compensate pixel values on the DLP. Because the resolution of the DLP was lower than that of the LCD, and because the DLP image sharpness was further reduced by a diffuser, it was necessary to model a point-spread function (PSF) of the DLP and to use it when factorizing target images into LCD and DLP components. The PSF was modeled by taking multiple exposures of the grid of dots, reconstructing from them an HDR image, and fitting a Gaussian function approximating the shape of the PSF.

The color calibration was performed by measuring display's spectral emission, individually for LCD and DLP, using a spectroradiometer (JETI Specbos 1211 in Cambridge, PhotoResearch PR-670 in Liverpool). CIE 2006 cone fundamentals (CIE, 2006) were used to calculate the L-, M-, and S-cone responses as follows:

(1) $$\begin{array}{ccc} L & = & 0.689903\int_{\lambda }l_{2}\left(\lambda \right)\;E\left(\lambda \right)\;d\lambda \;,\\ M & = & 0.348322\int_{\lambda }m_{2}\left(\lambda \right)\;E\left(\lambda \right)\;d\lambda \;,\\ S & = & 0.0371597\int_{\lambda }s_{2}\left(\lambda \right)\;E\left(\lambda \right)\;d\lambda \;,\; \end{array}$$

where *l*_2_, *m*_2_, and *s*_2_ are 2^○^ cone fundamentals^1^ and *E* is the measured spectral radiance emitted from the display. The *l*_2_ and *m*_2_ spectra were scaled such that the sum corresponded to luminance and the sensitivity of the S-cones was set so that *s*_2_(λ)/*V*(λ) peaks at 1 (CIE, 2006). All our calculations were based on photopic luminance, including the lowest luminance levels of 0.02 cd/m^2^, which was at the lower end of the mesopic range (Barbur & Stockman, 2010).

**Figure 2.**
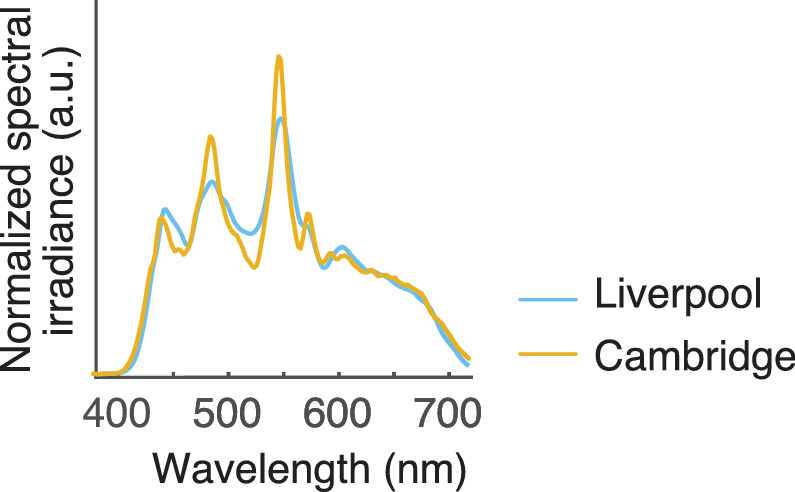
Spectral power distributions of the HDR displays.

The responses were fitted to the gain-offset-gamma display model (Berns, 1996) for the LCD and a 1-dimensional look-up table was used for the DLP (since it was achromatic after removing the color wheel); see Figure 2 for the spectral emission of the two HDR displays.

Both LCD and DLP were natively driven by 8-bit signals. To prevent banding artifacts from quantization, we used spatio-temporal dithering for LCD and bit-stealing for DLP to extend the effective bit-depth to 10-bits per color channel. The display driver was written in the OpenGL shading language to factorize and render images in real-time.

#### Stimuli

**Figure 3.**
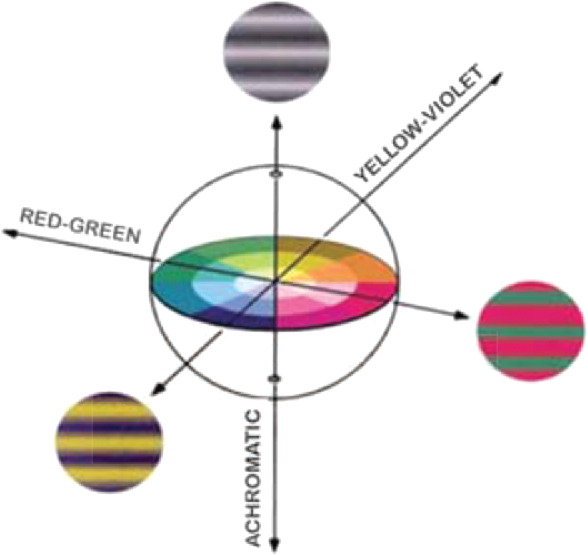
Color space with the three modulation directions used in the experiments.

**Figure 4.**
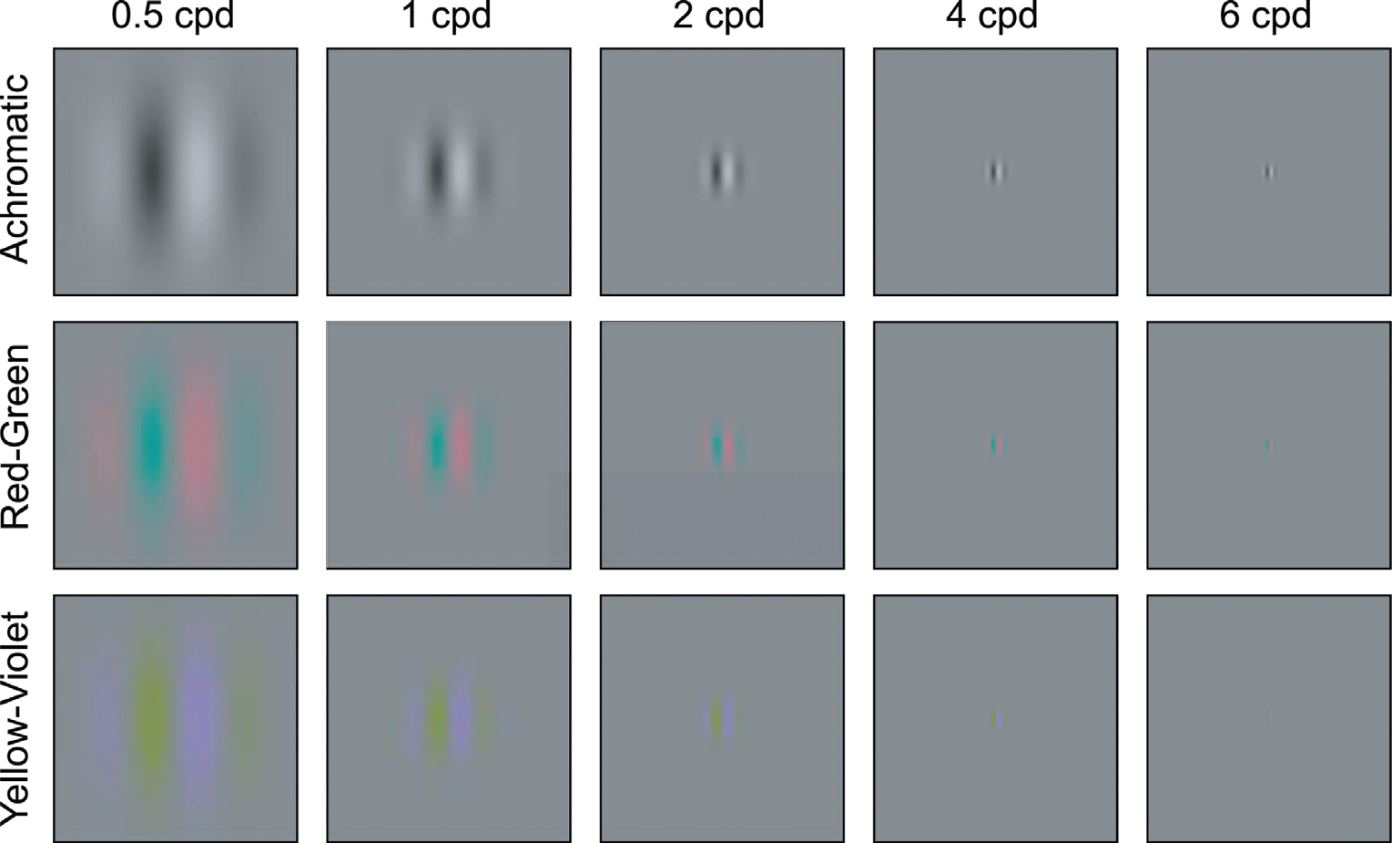
Fixed-cycles stimuli used in Experiments 1 to 3. The width of the Gaussian envelope was set to be half of the wavelength, σ = (0.5/*f*)^○^.

The stimuli were Gabor patches created by multiplying a sinusoidal grating with a Gaussian envelope (Figure 4). The Gabor were odd-symmetric, that is, the phase was adjusted so that the zero-crossing was exactly in the center of the stimulus. Each grating was modulated along one of the three cardinal color axes in Derrington-Krauskopf-Lennie (DKL) space (Figure 3): an achromatic, red-green, or yellow-violet direction (Derrington et al., 1984). Modulations in this color space can either be described by the stimulus properties reflecting the appearance (achromatic, red-green, yellow-violet) or by the chromatic properties of a set of hypothesized mechanisms that are isolated by these stimulus modulations (Brainard, 1996).

In terms of the stimulus properties, changes along the achromatic direction resulted in all three cone classes being modulated such that the cone contrasts are identical; modulations along the red-green axis leave the excitation of the S-cones constant, and the excitation of the L- and M-cones covaries as to keep their sum constant. Along the third, the yellow-violet direction, only the S-cones are modulated. These modulations in color space are designed to isolate a set of three hypothesized mechanisms: a luminance mechanism $\left(R_{L+M}\right)$ and two cone-opponent color mechanisms ($R_{L-M}$, $R_{S-(L+M)}$).

The chromatic properties are described in the matrix below (Equation 2). The first mechanism, $\left(R_{L+M}\right)$, is the luminance mechanism, which adds up the L- and M-cone responses (which are normalized such that the sum corresponds to *V*(λ)). The second mechanism, $\left(R_{L-M}\right)$, is an L/M opponent mechanism and takes the differences between the weighted incremental L- and M-cone signals. The third mechanism, $\left(R_{S-(L+M)}\right)$, is another cone-opponent mechanism taking the difference between the incremental S-cone signal and the sum of the incremental L- and M-cones.

(2) $$\begin{array}{ccc} \left[\begin{array}{c} \Delta R_{L+M}\\ \Delta R_{L-M}\\ \Delta R_{S-(L+M)} \end{array}\right] & = & \left[\begin{array}{ccc} 1 & 1 & 0\\ 1 & -\frac{L_{0}}{M_{0}} & 0\\ -1 & -1 & \frac{L_{0}+M_{0}}{S_{0}} \end{array}\right]\\ & & \times \;\left[\begin{array}{c} \Delta L\\ \Delta M\\ \Delta S \end{array}\right] \end{array}$$

where *L*_0_, *M*_0_, and *S*_0_ are the cone responses corresponding to the gray background. Stimuli were modulated around this neutral gray (white) background of a D65 metamer (CIE 1931 x, y = 0.3127, 0.3290).

The inverse of the above matrix defines the stimulus modulations in LMS space that are required to achieve selective stimulation of the hypothesized mechanisms and is shown below (Equation 3). For example, to isolate the luminance mechanism $\left(R_{L+M}\right)$, we set the mechanism output vector to [1 0 0], which results in changes in all three cone signals. To isolate the cone-opponent mechanism $\left(R_{L-M}\right)$, we set the response vector to [0 1 0], which results in equal L- and M-cone modulations but of opposite sign. Finally, to isolate the third opponent mechanism $\left(R_{S-(L+M)}\right)$, the response vector is set to [0 0 1], resulting only in S-cone modulations. The matrix that maps the mechanisms, output into the LMS modulations depends on the chromaticity of the background. Equation 4 shows the matrix used in our experiment. The desired LMS modulations can then be converted to linearized RGB (see the Acknowledgments for links to the MATLAB files). For a tutorial on how to implement the DKL space, the reader should consult Brainard (1996).

(3) $$\begin{array}{ccc} \left[\begin{array}{c} \Delta L\\ \Delta M\\ \Delta S \end{array}\right] & = & \left[\begin{array}{ccc} \frac{L_{0}}{L_{0}+M_{0}} & \frac{M_{0}}{L_{0}+M_{0}} & 0\\ \frac{M_{0}}{L_{0}+M_{0}} & -\frac{M_{0}}{L_{0}+M_{0}} & 0\\ \frac{S_{0}}{L_{0}+M_{0}} & 0 & \frac{S_{0}}{L_{0}+M_{0}} \end{array}\right]\\ & & \times \;\left[\begin{array}{c} \Delta R_{L+M}\\ \Delta R_{L-M}\\ \Delta R_{S-(L+M)} \end{array}\right] \end{array}$$

(4) $$\begin{array}{ccc} \left[\begin{array}{c} \Delta L\\ \Delta M\\ \Delta S \end{array}\right] & = & \left[\begin{array}{ccc} 0.6981 & 0.3019 & 0\\ 0.3019 & -0.3019 & 0\\ 0.0198 & 0 & 0.0198 \end{array}\right]\\ & & \times \;\left[\begin{array}{c} \Delta R_{L+M}\\ \Delta R_{L-M}\\ \Delta R_{S-(L+M)} \end{array}\right] \end{array}$$

To achieve comparable response units in these three mechanisms, the responses could be scaled such that the response for each mechanism is unity for a stimulus of unit pooled cone contrast. However, all these scaling procedures are to a large extent arbitrary (Capilla et al., 1998). We therefore used the length in cone contrast space (Equation 5) as a measure of stimulus contrast since it allows comparison across different color directions (Cole et al., 1993). The rationale for measuring contrast sensitivity along these three modulation directions in color space was twofold. First, these modulations were likely to preferentially stimulate early postreceptoral mechanisms. While it was unlikely that cortical mechanisms could be isolated with these color modulations (Shapley & Hawken, 2011), it still allowed us to characterize the contrast sensitivity for salient and, to some degree, independent mechanisms. Second, it constituted a device-independent definition of the chromatic stimulus modulations and allowed comparisons with previously obtained CSF measurements.

The standard deviation of the Gaussian envelope was set to be half of the wavelength ($\sigma =0.5\cdot \frac{1}{f}$ [deg]). The Gabors were of spatial frequencies 0.5, 1, 2, 4, or 6 cycles per degree of visual angle (cpd). Thus, the ± 2σ region of the Gabor patches subtended $4^{\circ }\;\times \;4^{\circ }\;$, $2^{\circ }\;\times \;2^{\circ }\;$, $1^{\circ }\;\times \;1^{\circ }\;$, $0.5^{\circ }\;\times \;0.5^{\circ }\;$, and $0.33^{\circ }\;\times \;0.33^{\circ }\;$, respectively. Using these Gabor stimuli with a fixed number of visible cycles allowed us to treat the width of the Gaussian as a fixed parameter. This was useful for modeling, since we could then treat the width of the Gaussian envelope as a free parameter for predicting contrast sensitivity to stimuli of different sizes.

#### Procedure

The experiment was grouped into multiple sessions by mean luminance level to ensure that observers were fully adapted to the display luminance during data collection. The mean luminance was one of 0.02, 0.2, 2, 20, 200, 2,000, or 7,000 cd/m^2^; assuming Watson's (2012) unified pupillary model, these luminances were equivalent to 0.86, 7.83, 62.87, 416.80, 2,335.85, 13,245.57, and 36,560.55 trolands, respectively. For sessions at 0.02 and 0.2 cd/m^2^, observers adapted to the darkness for 5 to 10 minutes prior to starting the study and remained in the experiment room until the end of the session. Sessions at 7,000 cd/m^2^ were conducted exclusively in Cambridge.

At the beginning of each session, we obtained a preliminary estimate of the contrast threshold using a method of adjustment task. This was used as an initial estimate for the QUEST procedure.

The main task was a 4AFC (four-alternive forced choice) detection task, in which observers indicated which quadrant of the display contained a Gabor patch. The stimulus was positioned 3.77^○^ from the center of the display: upper left, upper right, lower left, or lower right. The stimulus was displayed until observer response. Between trials, a mask was presented over the 4AFC stimulus region for 500 ms to neutralize adaptation to the previously seen Gabor. To create the mask, we sampled a matrix of random numbers from U (− 1, 1) per color channel, then blurred the resulting image with a Gaussian kernel (σ = 4 px).

The stimulus contrast was determined using a QUEST procedure (Watson & Pelli, 1983). There was one QUEST staircase per spatial frequency and color modulation combination, for a total of 21 staircases per session. Each staircase lasted for a minimum of 25 and a maximum of 35 trials.

Within a session, observers saw Gabor patches of different spatial frequencies and color modulation interleaved in a random order. Since the Gabor orientation was not a stimulus dimension of interest, we randomly chose a vertical or horizontal orientation for each trial. Observers had no information as to the spatial frequency, color modulation, or orientation of the target Gabor patch.

Each session lasted approximately 40 to 50 minutes. Some observers chose to omit sessions at 7,000 cd/m^2^, as the high luminance could be uncomfortable to view for an extended period of time.

Observers were seated 91 cm from the HDR display such that the display subtended $12.5^{\circ }\;\times \;9.4^{\circ }\;$. The effective sampling rate of the LCD was 165 pixels per visual degree. The head position was fixed with a chin-rest to the horizontal and vertical center of the display. Observers were allowed to move their eyes in order to examine stimuli. All viewing was binocular. Our rationale for unlimited viewing time and free scanning of the display was driven by two considerations. First, since our aim was to provide a model of contrast sensitivity applicable to everyday viewing conditions, unlimited viewing time seemed to be the most appropriate choice. Second, in parallel to the experiments reported here, we have been collecting data from observers falling into an older age group (60+ years old). For these observers, it is difficult to obtain robust data with very brief stimulus durations.

### Results

For each condition, we computed the maximum likelihood estimate of the contrast sensitivity. Each threshold estimate is typically based on between 25 and 35 trials. Threshold contrast is defined as the normalized length in cone contrast space (Equation 5):

(5) $$\begin{array}{ccc} & & \;C_{\text{t}}=\frac{1}{\sqrt{3}}\sqrt{{\left(\frac{\Delta L}{L_{0}}\right)}^{2}+{\left(\frac{\Delta M}{M_{0}}\right)}^{2}+{\left(\frac{\Delta S}{S_{0}}\right)}^{2}}\\ & & \;C_{\text{t}}=\text{Threshold}\;\text{cone}\;\text{contrast}\\ & & \;\Delta L,\Delta M,\Delta S=\;\text{Incremental}\;\text{L,M,S-cone}\\ & & \;\;\;\;\;\times \;\text{absorptions}\\ & & \;L_{0},M_{0},S_{0}=\text{L,M,S}\;\text{absorptions}\;\text{of}\;\text{the}\\ & & \;\;\;\;\;\;\;\times \;\text{display}\;\text{background}\; \end{array}$$

The advantage of this contrast measure is that it allows device-independent comparisons between different directions in color space and is identical to the standard Michelson contrast for achromatic modulations.

**Figure 5.**
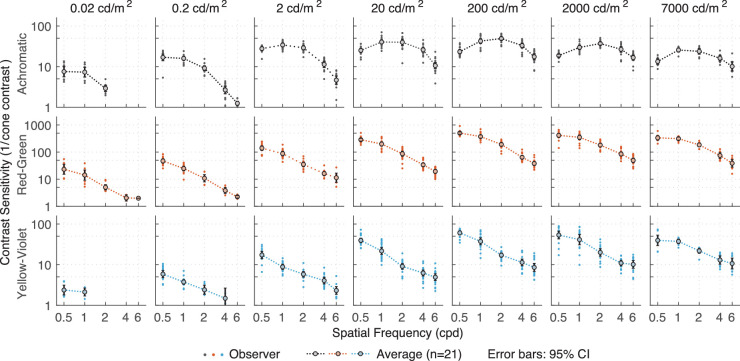
Results of Experiment 1. Contrast sensitivity as a function of luminance for the three color directions: achromatic, red-green, and yellow-violet.

Figure 5 shows the contrast sensitivities as a function of frequency for light levels ranging from 0.02 cd/m^2^ to 7,000 cd/m^2^. The achromatic modulations resulted in a classic band-pass response for medium to high luminance levels (from 2 cd/m^2^ onward), with a peak response at medium spatial frequencies (ranging from 1 to 2 cpd). The gradual change from a low-pass shape at very low luminance levels (0.02 cd/m^2^) to the typical band-pass shape in higher luminance levels is similar to the results of Van Nes and Bouman (1967). Red-green and yellow-violet modulations, on the other hand, resulted in a low-pass contrast sensitivity curves at all light levels, with the peak sensitivity occurring at the lowest spatial frequency measured (0.5 cpd). Sensitivity was higher for the red-green stimuli than for the achromatic modulation when expressed as the inverse of the cone contrast, which is consistent with Kim et al. (2017).

**Figure 6.**
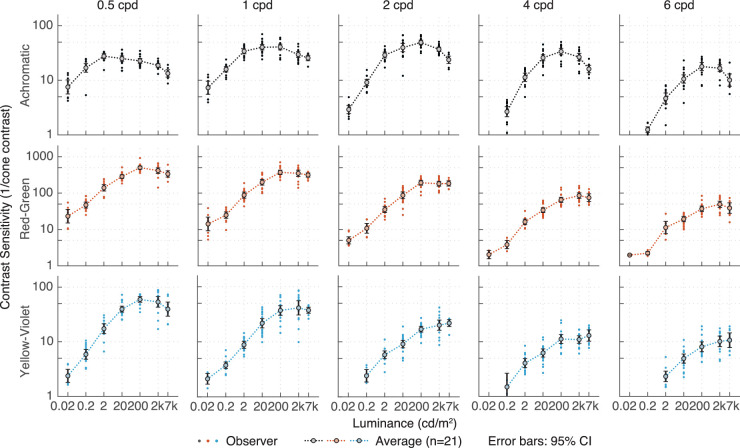
Contrast sensitivity replotted from Figure 5 as a function of luminance.

When contrast sensitivity data were replotted as a function of light level (Figure 6), sensitivity was not a monotonic function of luminance for achromatic modulations; rather, contrast sensitivity was lowest at 0.02 cd/m^2^ and rose steadily with increasing mean luminance until it reached a peak at 20 to 200 cd/m^2^ for low to medium frequencies, then decreased again beyond 200 cd/m^2^. This luminance dependence interacted with spatial frequency, such that the overall maximum sensitivity occurred between 20 to 200 cd/m^2^ for 1 to 2 cpd where observers could reliably detect a Gabor patch of 2% to 3% contrast. For red-green and yellow-violet modulations, contrast sensitivity rose steadily as a function of luminance, reaching a maximum at around 200 cd/m^2^. Only for the lowest frequency, a decrease in peak sensitivity was observed.

**Figure 7.**
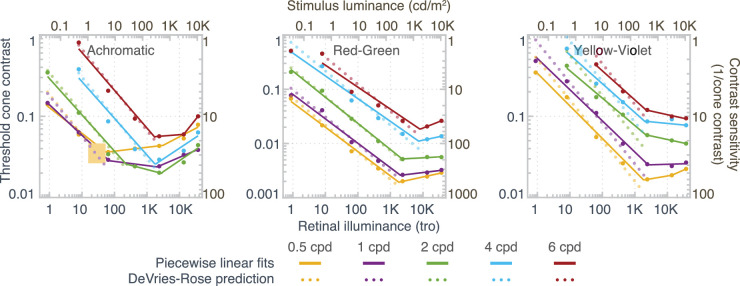
Logarithmic threshold cone contrast sensitivity as a function of log retinal illuminance.

In Figure 7, thresholds are plotted as a function of retinal illuminance (trolands). For chromatic stimuli (red-green and yellow-violet), contrast thresholds were independent of the retinal illuminance beyond about 2,000 trolands, hence consistent with Weber law, whereas for achromatic stimuli (L+M), thresholds rose again for very high light levels. This failure of Weber-law behavior in the high photopic range has not been reported by Van Nes and Bouman (1967), probably due to the fact that that they only investigated contrast sensitivity up to 5,900 trolands and our data show that Weber law only fails at retinal illuminances above 10,000 trolands.

For all three modulation directions, log threshold contrast decreased approximately linearly with log retinal illuminance for low and intermediate light levels, with slopes systematically a bit less than −0.5, (DeVries-Rose law; Rose, 1948; De Vries, 1943). Mean slopes were −0.42 and −0.36 for red-green and yellow-violet, respectively (Table 1) and independent of spatial frequency. For achromatic thresholds, the slopes were frequency dependent and increased with spatial frequency (Table 1), consistent with Mustonen et al. (1993).

**Table 1.** Slopes of log threshold contrast versus log retinal illuminance (trolands) in linear range.

	**Spatial frequency (cpd)**					
**Modulation**	**0.5**	**1**	**2**	**4**	**6**	**Mean**
Achromatic	−0.31259	−0.37537	−0.42091	−0.43269	−0.4546	−0.39923
Red-green	−0.43583	−0.42582	−0.46969	−0.38018	−0.40045	−0.42239
Yellow-violet	−0.37897	−0.37221	−0.34183	−0.35667	−0.35517	−0.36097

The transition from the DeVries-Rose to Weber behavior was independent of spatial frequency for chromatic modulations (Figure 7); for achromatic stimuli, on the other hand, the inflection point shifted to higher retinal illuminances when spatial frequency was increased. Díez-Ajenjo and Capilla (2010) and Valero et al. (2004) reported a similar difference between chromatic and achromatic gratings: For achromatic gratings, the transition from DeVries-Rose to Weber-law behavior was dependent on spatial frequency and occurred between 1 and 2  cd/m^2^ for the lowest spatial frequency measured (0.5 cpd), consistent with our findings. For chromatic modulations, threshold contrast decreased approximately linearly with background luminance in log-log space, without a clear transition point up to 100 cd/m^2^. Valero et al. (2004) only investigated luminances up to 100  cd/m^2^, which is well below our maximum luminance range (7,000  cd/m^2^); in our experiments (Figure 7), the transition point occurred at around 200  cd/m^2^ for chromatic stimuli.

The failure of Weber law behavior for very high luminances may be due to incomplete adaptation to the display background for luminances greater than 200 cd/m^2^. We investigate this possibility in Experiment 2, presented in the following section.

## Experiment 2: Control for incomplete adaptation

The purpose of Experiment 2 was to determine whether incomplete adaptation to the mean luminance level affected the contrast sensitivity measurements at high luminances ($>200\;{\text{cd/m}}^{2}$). Though luminance adaptation is largely local and typically limited to a 0.5^○^-radius neighborhood (Vangorp et al., 2015), the adaptation level can nonetheless be influenced by more distant parts of the visual field. As Experiment 1 was conducted in a dark room and the display subtended only a small portion of the visual field, we considered the possibility that the dark surroundings prevented observers from becoming fully adapted to the high luminance of the display.

Our hypothesis was that such incomplete adaptation was responsible for the drop in sensitivity that we observed at luminance levels above 200 cd/m^2^. To test this hypothesis, we measured contrast sensitivities in bright surroundings. We kept the room light on and placed additional light sources around the display, in order to reduce the difference between the mean luminance of the display and of the region surrounding the display.

### Methods

Contrast sensitivity was measured at 7,000 cd/m^2^. Four observers (three female, one male, mean age = 29.0 ± 8.2) participated; two were authors. The stimuli and the apparatus were identical to those in Experiment 1.

In addition to the HDR display, we placed two photographer's softboxes near the display, with the goal of increasing the luminance of the region surrounding the HDR display as uniformly as possible. Each softbox was fitted with five 5,500K CFL bulbs and enclosed with a white fabric diffuser. From the observer's perspective, one softbox was directly above the display and one was directly to the right. Due to space restrictions, we did not place any to the observer's left. The softboxes added 1,000 lux of light as measured from the observer's viewing position with a handheld digital light meter.

### Results

**Figure 8.**
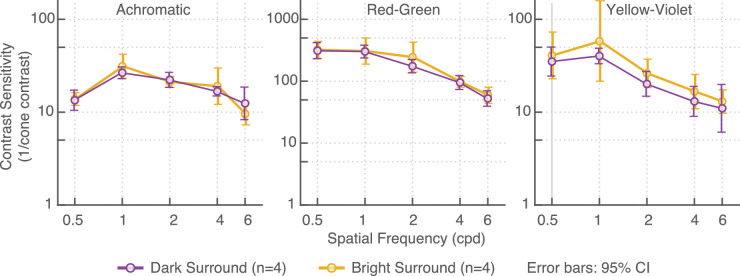
Contrast sensitivity measures in dark (dark symbols) and bright (bright symbols) surroundings. In the dark surround condition, only the HDR display emitted light (7,000 cd/m^2^). No systematic differences were found between these two conditions.

For the stimulus conditions tested, we did not find any systematic differences in contrast sensitivity when observers were in a dark room or in a bright room with high ambient light levels (Figure 8). This suggests that incomplete adaptation alone cannot explain the drop in sensitivity at the luminance levels above 200 cd/m^2^.

## Experiment 3: Low spatial frequencies

In Experiments 1 and 2, contrast sensitivity for the red-green and yellow-violet modulations was low-pass in shape, that is, the peak sensitivity occurred at the lowest spatial frequency measured. In Experiment 3, we examined whether chromatic contrast sensitivity measurements at extremely low spatial frequencies would reveal a bandpass shape as observed for achromatic modulations. We therefore tested additional low frequencies ranging from 0.125 cpd to 6 cpd, at three luminance levels: 0.02, 200, and 7,000 cd/m^2^, for red-green and lime-violet stimuli.

### Methods

Five observers (two male, three female, mean age = 27.2 ± 4.3) from Cambridge and Liverpool participated in this experiment. One observer was naive; the rest were authors or had previously participated in Experiments 1 or 2. Two observers participated in the full set of spatial frequency conditions; the remaining three participated only in the three lowest spatial frequency conditions.

All stimulus parameters were as described in Experiment 1, but thresholds were only measured for the two chromatic directions. For the 0.125 cpd, 0.25 cpd, and 0.5 cpd conditions, observers were seated at 45.5 cm, such that the HDR display subtended $24.8^{\circ }\;\times \;18.7^{\circ }\;$ and could show up to four $9.0^{\circ }\;\times \;9.0^{\circ }\;$ Gabor patches at a time. Observers did not see a sharp boundary at the border of the $9^{\circ }\;\times \;9^{\circ }\;$ region, since the experiment was conducted near the observers’ contrast detection threshold.

### Results

**Figure 9.**
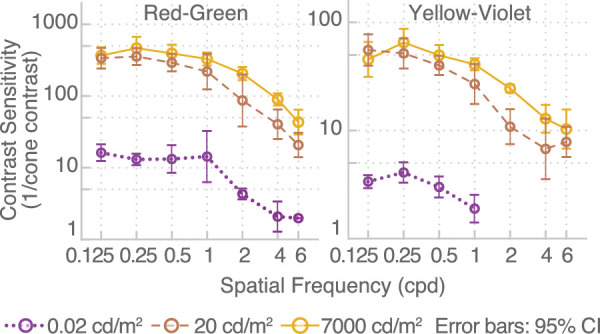
Chromatic contrast sensitivity extended to lower spatial frequencies from 0.125 cpd to 6 cpd.

We did not find a systematic reduction in contrast sensitivity at the very low frequency (0.125 cpd) for the low and intermediate (0.02 and 20 cd/m^2^) luminance levels (Figure 9). For the highest luminances (7,000 cd/m^2^), there was some evidence that the chromatic contrast sensitivity drops off as the achromatic sensitivity does. However, these differences are within measurement error, and our experiments do not provide any strong evidence against the low-pass characteristics of the chromatic contrast sensitivity.

## Experiment 4: Effect of stimulus size

The contrast sensitivity for periodic stimuli is known to depend on the number of cycles displayed (Hoekstra et al., 1974). Gratings with fewer cycles result in higher contrast thresholds, suggesting summation across cycles and/or spatial extent (Howell & Hess, 1978) until a critical summation area has been reached (Piper, 1903). Effect of stimulus area and number of cycles has been studied both in the fovea and the periphery, primarily for achromatic gratings (Manahilov et al., 2001). Studies using chromatic stimuli reported subthreshold spatial summation to be similar for achromatic and red-green gratings (Sekiguchi et al., 1993) but show a different dependence on eccentricity (Mullen, 1991) and larger integration areas for S-cone isolating gratings (Vassilev et al., 2000). The purpose of this additional experiment was to enable us to predict contrast sensitivity for stimuli of different sizes from our fixed-cycles data.

### Methods

In Experiment 1, the Gaussian envelope size was equal to half wavelength, where wavelength is the inverse of spatial frequency. For the current experiment, we introduced two more envelope sizes equivalent to one and two wavelengths, respectively. This manipulation allowed us to investigate spatial summation for each spatial frequency since contrast sensitivity was measured for three different envelope sizes. This experiment was conducted at 20  cd/m^2^ and only with a subset of the observers of Experiment 1, namely, 11 observers from Cambridge and Liverpool (four male, seven female, mean age = 30.7 ± 11.9). The procedure and apparatus were identical to Experiment 1.

### Results

**Figure 10.**
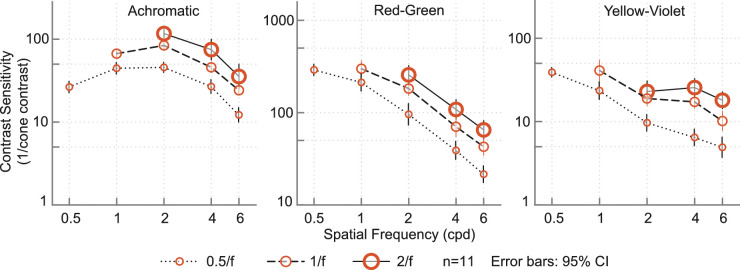
Results of Experiment 4: Each line represents the contrast sensitivity function for a series of stimuli with different number of cycles and consequently different stimuli sizes. The size of the Gaussian envelope was fixed to 0.5, 1, and 2 times the wavelength (the inverse of spatial frequency).

**Figure 11.**
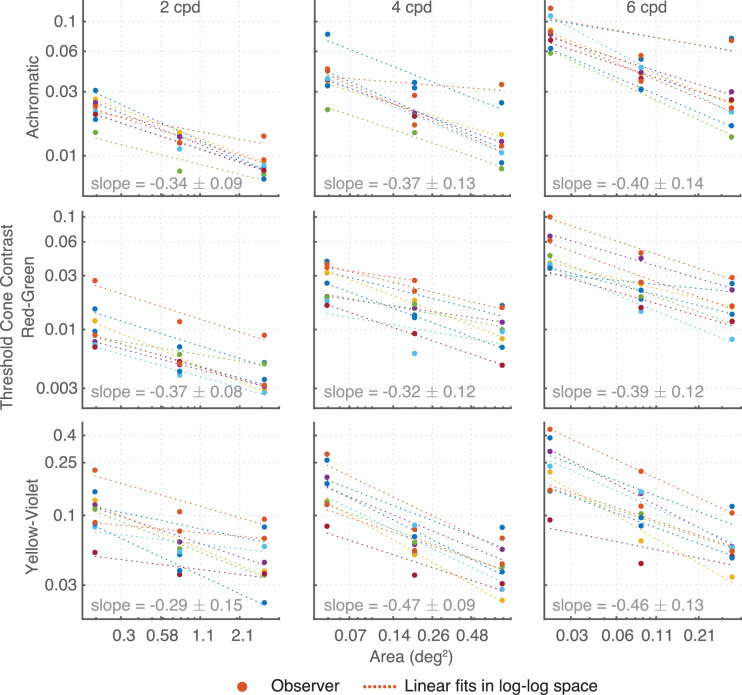
Linear decrease in log contrast with increase in log area of the stimulus.

Contrast sensitivity increased with stimulus size (Figure 10). Due to display size restrictions, not all spatial frequencies could be measured at all three envelope sizes. However, the available data suggest that an increase in envelope size causes a fixed increase in sensitivity in log-log space. In Figure 11, contrast thresholds are replotted as a function of area for three different frequencies (2, 4, 6 cpd) with slopes in log-log space varying from −0.29 to −0.47. Slopes of −0.5 are consistent with Piper's law (Luntinen et al., 1995) and can be modeled as a single-filter contrast energy model (Manahilov et al., 2001); slopes in the region from −0.25 to −0.5 reflect probability summation between multiple filters or nonlinear summation mechanisms (Meese & Summers, 2007). We return to the dependency on stimulus size in the modeling section.

## Modeling

**Figure 12.**
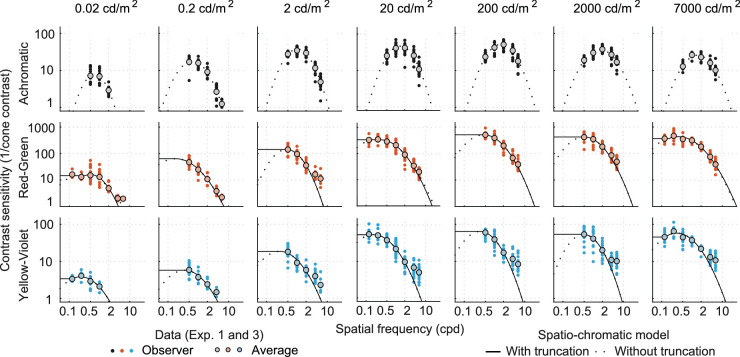
The results of fitting parabolic CSF models to the data, individually for each luminance level (columns) and color direction (rows). Note that the frequencies below 0.5 cpd were measured only at 20 cd/m^2^ and for the chromatic color channels.

Our goal was to derive a spatio-chromatic contrast sensitivity function that could interpolate and extrapolate the collected data within an allowable range. We constructed a set of nested models, with each successive model being more restrictive and with fewer free parameters. In Model 1 (“spatio-chromatic contrast sensitivity function”), the CSF was fitted separately for each color direction and each luminance level (each panel in Figure 12 is fitted separately). Model 2 (including “luminance intrusion”) restricts the fits by assuming that the CSF for chromatic stimuli is a mixture of a purely chromatic CSF and a luminance CSF for high spatial frequencies. In Model 3, a functional relationship between the model parameters and the adapting light level (“CSF as a function of adapting light level”) was introduced.

Subsequently, contrast sensitivity measurements for different envelope sizes were used to generalize the model predictions from fixed-cycles stimuli to stimuli of arbitrary sizes (“CSF as the function of the stimulus size”) and the extended model was used to predict previously published contrast sensitivity data (Mantiuk et al., 2011; Kim et al., 2013; Wuerger et al., 2002).

### Spatio-chromatic contrast sensitivity function

As a function of spatial frequency, the achromatic CSF is band-pass and the chromatic CSFs have a low-pass shape (Figures 5 and 9). We modeled this behavior using a truncated log-parabola (Ahumada & Peterson, 1992; Rohaly & Owsley, 1993; Watson & Ahumada, 2005; Kim et al., 2017):

(6a) $$\begin{array}{ccc} & & \;\log_{10}S\left(f;S_{\text{max}},f_{\text{max}},b\right)\\ & & \;\;=\log_{10}S_{\text{max}}-{\left(\frac{\log_{10}f-\log_{10}f_{\text{max}}}{0.5\cdot 2^{b}}\right)}^{2}\; \end{array}$$

(6b) $$\begin{array}{ccc} & & \;S^{'}\left(f;S_{\text{max}},f_{\text{max}},b,t\right)\\ & & \;=\left\{\begin{array}{cc} \frac{S_{\text{max}}}{t}, & \text{if}\;f<f_{\text{max}}\;\text{and}\;S\left(f;S_{\text{max}},f_{\text{max}},b\right)<\frac{S_{\text{max}}}{t}\\ S(f) & \text{otherwise} \end{array}\right.\\ \; \end{array}$$

Equation 6 has four parameters: peak frequency *f*_max_, peak sensitivity *S*_max_, bandwidth *b*, and an optional truncation parameter *t*. *t* describes the low-pass behavior in sensitivity functions where the sensitivity saturates to a constant value for spatial frequencies below the peak frequency.

We first model all CSFs as log-parabola without the truncation parameter and then model the chromatic CSFs as truncated log-parabolas. The three color channels and the seven luminance levels are modeled independent of each other. We fitted the average data for each of the 21 conditions (seven luminances and three color channels) with either three (*f*_max_,*S*_max_,*b*) or four (*f*_max_,*S*_max_,*b*,*t*) free parameters.

We made the implicit assumption that the contrast sensitivity of the chromatic stimulus modulations (red-green, yellow-violet) is determined by the sensitivity of two putative chromatic mechanisms. While chromatic mechanisms favor low temporal and low spatial frequencies, it is unlikely that chromatic contrast variations at medium to high frequencies (4 and 6 cpd) are only seen by chromatic mechanisms (due to luminance artifacts; see Introduction for details). Based on the data from Mullen (1985), we fitted the nominally isoluminant chromatic data using only the spatial frequencies ≤ 2 cpd.

The results are in Figure 12 and Table 2. The log-parabola model fits the achromatic data well, but a truncated log-parabola model is needed to explain the chromatic data, especially at the lower frequencies, which were measured only at 20 cd/m^2^. The chromatic data show a small dip in sensitivity at the extreme luminance levels of 0.02 cd/m^2^ and 7,000 cd/m^2^. At this stage, we cannot confirm whether the dip reflects a real effect or measurement error.

**Table 2.** Parameters for log-parabola fit with truncation parameter for chromatic channels.

		**Luminance ( cd/m^2^)**						
**Parameter**	**Channel**	**0.02**	**0.2**	**2**	**20**	**200**	**2,000**	**7,000**
*f*_max_	Achromatic	0.6839	0.6371	1.023	1.372	1.624	1.689	1.540
	Red-green	0.5704	0.2596	0.4536	0.3094	0.4422	0.5547	0.5501
	Yellow-violet	0.2702	0.4407	0.3543	0.1679	0.3344	0.4783	0.3263
*S*_max_	Achromatic	7.825	17.63	37.45	46.46	50.89	36.44	25.80
	Red-green	15.73	53.93	142.6	347.8	508.9	417.4	388.6
	Yellow-violet	3.845	5.536	17.16	54.57	64.42	53.69	57.93
*b*	Achromatic	0.7809	0.9883	0.903	0.9082	0.9475	1.064	1.003
	Red-green	0.8471	1.153	0.9108	1.17	1.123	1.015	1.055
	Yellow-violet	1.159	1.156	1.155	1.356	1.126	1.041	1.271
*t*	Red-green	0.0339	0.000	0.000	0.0132	0.000	0.0024	0.000
	Yellow-violet	0.0576	0.000	0.000	0.000	0.000	0.000	0.1048

### Luminance intrusion

The CSF model in Figure 12 predicted lower sensitivities for the chromatic modulations (R-G, Y-V) at frequencies greater than 4 cpd than what we found in the experiments. We hypothesized that this was caused by the intrusion of a luminance mechanism at higher spatial frequencies (Flitcroft, 1989), possibly because we did not make the stimuli isoluminant for each observer using heterochromatic flicker photometry. We modeled this luminance intrusion by predicting chromatic sensitivity as the combination of responses of both luminance and chromatic mechanisms.

The probability that a stimulus defined by color contrast will be detected by achromatic or chromatic channels can be modeled as probability summation:

(7) $$\begin{array}{ccc} & & \;P_{Ach+Chr}=1-\left(1-P(\alpha \;C\;S_{\text{Ach}})\right)\left(1-P(C\;S_{\text{Chr}})\right) \end{array}$$

where *P*_Ach + Chr_ is the probability of detecting stimulus of the contrast *C*, *S*_Ach_ is the sensitivity of the achromatic channel, and *S*_Chr_ is the sensitivity of one of the chromatic channels (either red-green or yellow-violet). α is the portion of the original contrast that is detected by the luminance mechanism. Note that the product $C\;S_{\text{Ach}}$ gives the perceptually “normalized” contrast that is equal to 1 at the detection threshold. The function *P*(*c*) is the psychometric function that can be expressed as

(8) $$P\left(c\right)=1-\exp\left(\tau \;c^{\beta }\right)\;,$$

where β controls the slope of the psychometric function and τ controls the probability at the detection threshold. Since the thresholds were estimated from the 4AFC data for *P* = 0.81, we set τ to ln (0.81). If we introduce the psychometric function to Equation 7, we get

(9) $$\begin{array}{ccc} & & \;P_{\text{Ach+Chr}}=1-\exp\left(\tau {\left(\alpha \;C\;S_{\text{Ach}}\right)}^{\beta })\right)\exp\left(\tau {\left(C\;S_{\text{Chr}}\right)}^{\beta }\right) \end{array}$$

(10) $$=1-\exp\left(\tau \;C^{\beta }\left(\alpha^{\beta }\;S_{\text{Ach}}^{\beta }+S_{\text{Chr}}^{\beta }\right)\right)$$

If we introduce the psychometric function on the left side of the equation, we get

(11) $$\begin{array}{ccc} & & \;1-\exp(\tau \;C^{\beta }\;S_{\text{Ach+Chr}}^{\beta })\\ & & \;\;=1-\exp\left(\tau \;C^{\beta }\left(\alpha^{\beta }\;S_{\text{Ach}}^{\beta }+S_{\text{Chr}}^{\beta }\right)\right)\; \end{array}$$

(12) $$S_{\text{Ach+Chr}}={\left(\alpha^{\beta }\;S_{\text{Ach}}^{\beta }+S_{\text{Chr}}^{\beta })\right)}^{1/\beta }$$

Therefore, the sensitivity for the combined response of the chromatic and achromatic channels can be modeled as a weighted Minkowski summation of the sensitivities of the individual mechanisms.

**Figure 13.**
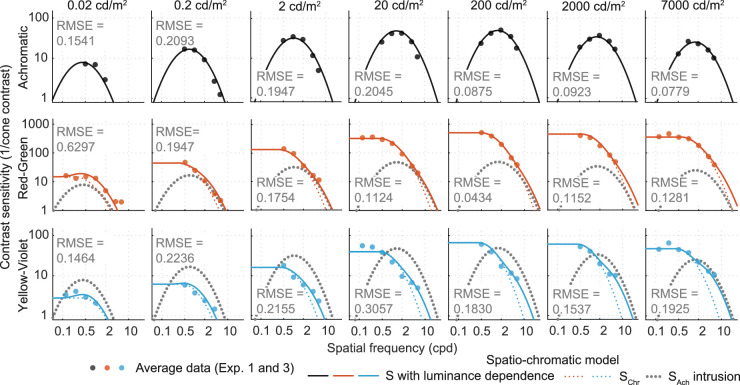
Channel summation model with 11 free parameters; see Table 3 for fitted parameters. Including luminance intrusion improves the model prediction for chromatic channels at higher frequencies. Filled dots represent the measured data for contrast sensitivities. Solid lines are the resultant model predictions while the dotted lines in cases of chromatic contrast sensitivities represent the pure chromatic and the luminance intrusion components.

The achromatic sensitivity is modeled using the log-parabola model from Equation 6:

(13) $$S_{\text{Ach}}=S\left(f;f_{\text{max}}^{\text{(Ach)}},S_{\text{max}}^{\text{(Ach)}},b^{\text{(Ach)}}\right)$$

where $f_{\text{max}}^{\text{(Ach)}}$, $S_{\text{max}}^{\text{(Ach)}}$, and *b*^(Ach)^ are the peak frequency, peak sensitivity, and bandwidth of the achromatic channel, at a given luminance level. The sensitivity to the two chromatic directions is modeled as the Minkowski summation of both chromatic and achromatic sensitivity:

(14) $$\begin{array}{ccc} & & \;S_{\text{Ach+RG}}=\left(\alpha_{RG}^{\beta }S_{Ach}^{\beta }\left(f;f_{\text{max}}^{\text{(Ach)}},S_{\text{max}}^{\text{(Ach)}},b^{\text{(Ach)}}\right)\right.\\ & & \;\;{\left.+\;S_{RG}^{'\beta }\left(f;f_{\text{max}}^{\text{(RG)}},S_{\text{max}}^{\text{(RG)}},b^{\text{(RG)}},t^{\text{(RG)}}\right)\right)}^{1/\beta }\; \end{array}$$

(15) $$\begin{array}{ccc} & & \;S_{\text{Ach+YV}}=\left(\alpha_{\text{YV}}^{\beta }S_{Ach}^{\beta }\left(f;f_{\text{max}}^{\text{(Ach)}},S_{\text{max}}^{\text{(Ach)}},b^{\text{(Ach)}}\right)\right.\\ & & \;\;{\left.+\;S_{YV}^{'\beta }\left(f;f_{\text{max}}^{\text{(YV)}},S_{\text{max}}^{\text{(YV)}},b^{\text{(YV)}},t^{\text{(YV)}}\right)\right)}^{1/\beta }\; \end{array}$$

where $f_{\text{max}}^{\text{(RG)}}$, $S_{\text{max}}^{\text{(RG)}}$, *b*^(RG)^, *t*^(RG)^, $f_{\text{max}}^{\text{(YV)}}$, $S_{\text{max}}^{\text{(YV)}}$, *b*^(YV)^, and *t*^(YV)^ are the parameters of the two chromatic mechanisms, fitted independently for each luminance level. The parameters α_RG_ and α_YV_ control the amount of luminance intrusion. At each luminance level, we fit all three sensitivity functions, 13 parameters in total (three peak frequencies, three peak sensitivities, three bandwidths, two summation coefficients, two achromatic channel gains). The optimization was performed for the data of all 20 observers individually as well as the average CSF for all the observers. The fitting results for the average CSF data are presented in Figure 13. The log-parabola fits (truncated in cases of chromatic channels) are shown as dotted lines in Figure 13. The model assumes that the achromatic stimuli are picked up solely by a luminance channel (upper row) and can be completely specified by Equation 13. For chromatic stimuli, we assumed that a luminance channel also contributes to the overall contrast sensitivity. In the second and third rows in Figure 13, the dotted lines represent the contributing luminance channel, which adds to the chromatic sensitivity via probability summation (Equation 7) and determines the response at higher spatial frequencies. The effect is more evident for the lime-violet stimuli.

The fitted parameters for the model are listed in Table 3. The values for α_RG_ are much higher than for α_YV_, which is due to the sensitivity values for red-green being higher than for yellow-violet or achromatic channels. This difference in sensitivity is partly due to the way contrast is defined (Equation 5). A quick investigation of the table reveals that many of the parameters are related to the logarithmic value of luminance. In the next section, we model such a functional relationship so that the model can be generalized to any luminance level within the measured range.

**Table 3.** Parameters for channel summation fit.

		**Luminance ( cd/m^2^)**						
**Parameter**	**Channel**	**0.02**	**0.2**	**2**	**20**	**200**	**2,000**	**7,000**
*f*_max_	Achromatic	0.5052	0.6368	1.016	1.349	1.652	1.701	1.547
	Red-green	0.4735	0.2907	0.3889	0.3690	0.5028	0.5506	0.5622
	Yellow-violet	0.2463	0.5571	0.5226	0.2410	0.3849	0.4831	0.4314
*S*_max_	Achromatic	7.138	17.63	37.29	41.43	47.29	36.02	25.16
	Red-green	14.44	45.85	128.3	335.4	501.6	415.6	387.3
	Yellow-violet	3.595	4.973	13.60	52.53	63.39	54.09	51.43
*b*	Achromatic	1.158	0.9886	0.9086	1.02	1.025	1.08	1.031
	Red-green	0.9825	1.221	1.201	1.052	1.016	1.023	1.038
	Yellow-violet	1.055	1.216	1.274	1.067	0.9617	0.9754	1.029
α	Red-green	2.858	1.089	1.315	1.037	1.527	2.750	3.120
	Yellow-violet	0.3480	0.2646	0.2672	0.2443	0.3513	0.5305	0.8683

### Contrast sensitivity as a function of mean luminance

**Figure 14.**
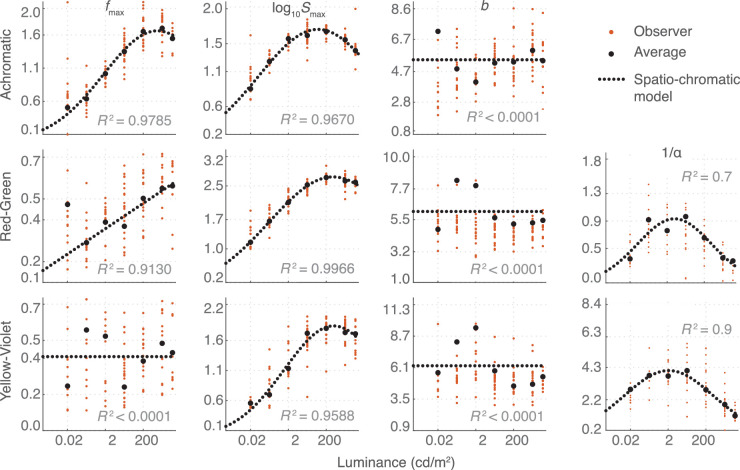
The relationship between the fitted CSF parameters and luminance. The orange dots indicate parameters fitted for individual observers and the black dots the parameters fitted for the average observer. The dashed lines show the functions we fitted to the parameters from average observer data to build a luminance-dependent CSF. The adjusted *R*^2^ values of the fits to the average observer are reported. *b* (in octaves) for all channels and *f*_max_ for the lime-violet channel did not fit well to a simple function and were thus fixed to the median value across luminance levels. Left: Log-parabola parameters; peak frequency *f*_max_, peak sensitivity *S*_max_, and bandwidth *b*. Right: Achromatic channel gain α used in Minkowski summation.

Figure 14 shows the relationship between the fitted CSF parameters and the logarithmic luminance. The plots clearly show that some parameters, such as *f*_max_, *S*_max_, and the inverse of α, are strongly related to log-luminance, while the relation of *b* is less clear given our data. To be able to generalize our model to different luminance levels (between 0.02 cd/m^2^ and 7,000 cd/m^2^), we fit functions for the CSF parameters that show strong relationship with luminance and find constant values for the parameter *b*, as listed in the equations below:

(16a, b) $$\begin{array}{ccc} & & \;f_{\text{max}}=\left\{\begin{array}{cc} 1.663\phi (\log l;3.045,2.834), & Achromatic\\ 0.06069\log l+0.3394, & Red\text{-}green\\ 0.4095 & Yellow\text{-}violet \end{array}\right.\\ & & \;\log_{10}S_{\text{max}}=\left\{\begin{array}{cc} 1.705\phi (\log l;1.867,3.142), & Achromatic\\ 2.715\phi (\log l;2.663,3.364), & Red\text{-}green\\ 1.843\phi (\log l;2.696,2.608), & Yellow\text{-}violet \end{array}\right. \end{array}$$

(16c, d) $$\begin{array}{ccc} & & \;b=\left\{\begin{array}{cc} 1.036 & Achromatic\\ 1.085 & Red\text{-}green\\ 1.097 & Yellow\text{-}violet \end{array}\right.\\ & & \;\frac{1}{\alpha }=\left\{\begin{array}{cc} 0.9323\phi (\log l;0.6986,1.998), & Red\text{-}green\\ 4.099\phi (\log l;0.3328,2.336), & Yellow\text{-}violet \end{array}\right. \end{array}$$

where ϕ is a Gaussian function: $\phi \left(x;\mu ,\sigma \right)=\exp\left(\frac{-{\left(x-\mu \right)}^{2}}{2\;\sigma^{2}}\right)$.

**Figure 15.**
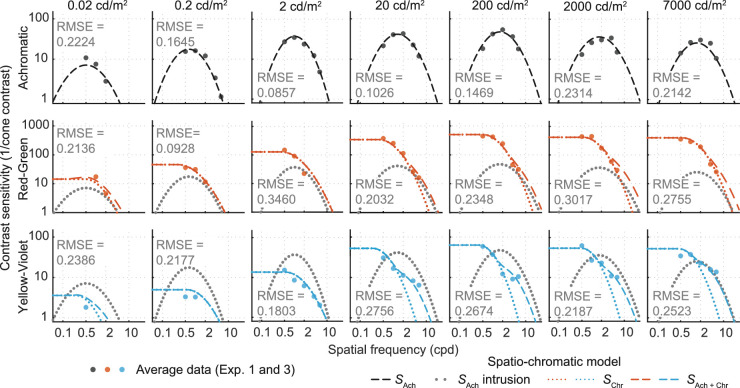
Model predictions including luminance intrusion and parameters as a function of the light level, based on Equations 13 to 16.

The summation coefficient β was fixed to 3.5. Figure 15 shows model predictions for the achromatic (Equation 13) and two chromatic (Equations 14 and 15) components of the model when the parameters are predicted by the functions and constants from Equation 16 *Yellow-violet*. Despite the approximations made to predict luminance-dependent parameters, the model provides good fit to the data.

The three models and their root-mean-squared-error (RMSE) are compared in Table 4. Model 1 was fitted individually for each measured luminance level and color direction. Model 2 was fitted for each luminance level but jointly for all color directions. Model 3 was fitted for seven luminance-dependent parameters and can generalize predictions to any arbitrary luminance level at the cost of higher RMSE.

**Table 4.** Summary of nested models.

**Model No.**	**Model description**	**Summary**	**Equations**	**Mean RMSE**	
1	Log-parabola	Optimization with three free parameters for Ach: $f_{\text{max}}^{\left(Ach\right)}$, $S_{\text{max}}^{\left(Ach\right)}$, *b*^(*Ach*)^; four free parameters for RG: $f_{\text{max}}^{\left(RG\right)}$, $S_{\text{max}}^{\left(RG\right)}$, *b*^(*RG*)^, *t*^(*RG*)^; and four free parameters for YV: $f_{\text{max}}^{\left(YV\right)}$, $S_{\text{max}}^{\left(YV\right)}$, *b*^(*YV*)^, *t*^(*YV*)^	Equation 6 fitted separately for each color and luminance	*Achromatic*	0.0463
				*Red-green*	0.0347
				*Yellow-violet*	0.0529
2	Model 1 + luminance intrusion	Optimization with 13 free parameters: $f_{\text{max}}^{\left(Ach\right)}$, $S_{\text{max}}^{\left(Ach\right)}$, *b*^(*Ach*)^, $f_{\text{max}}^{\left(RG\right)}$, $S_{\text{max}}^{\left(RG\right)}$, *b*^(*RG*)^, $f_{\text{max}}^{\left(YV\right)}$, $S_{\text{max}}^{\left(YV\right)}$, *b*^(*YV*)^, α^*RG*^, α^*YV*^, β^*RG*^, β^*YV*^ and two fixed parameters: *t*^(*RG*)^, *t*^(*YV*)^	Equations 13 – 15 fitted simultaneously for all colors, independently for each luminance	*Achromatic*	0.0701
				*Red-green*	0.1155
				*Yellow-violet*	0.1256
3	Models 1 + 2 + luminance dependence	Coefficients in Equation 16 optimized with three free parameters (Gaussian) and two free parameters (linear)	Equations 13 – 15 with parameters from Equation 16	*Achromatic*	0.1458
				*Red-green*	0.1998
				*Yellow-violet*	0.2029

### Contrast sensitivity as a function of stimulus size

When measuring stimuli of different frequencies, we fixed the number of cycles. This made the stimulus size become smaller as frequency increased. We had decided upon this approach in order to collect more applicable data in most applications, it is more important to know the exact threshold of a small pattern of high frequency rather than a large field of a high-frequency sine grating. But this choice also made our data harder to compare with other measurements, which were mostly done for stimuli of fixed size. In this section, we describe a model that can generalize our predictions to stimuli of arbitrary size and frequency so that model predictions can be compared with other data sets.

Rovamo et al. (1993) modeled spatial integration as a function that increases with the stimulus area and saturates after reaching a critical area. The key observation they made was that the increase in sensitivity is proportional to the square root of the product of grating area and the squared frequency. We follow their model but use the log-parabola sensitivity function rather than the OTF (Optical Transfer Function) used in the original paper:

(17) $$\begin{array}{ccc} & & \;S_{A}\left(f,a;S_{\text{max}},f_{\text{max}},b,a_{0},f_{0}\right)\\ & & \;\;=S\left(f;S_{\text{max}},f_{\text{max}},b\right)\cdot \sqrt{\frac{a\;f^{2}}{a_{0}+a\;f_{0}+a\;f^{2}}}\;,\;\; \end{array}$$

where *S*(*f*) is the log-parabola model from Equation 6, *f* is the spatial frequency in cycles per degree, and *a* is the area in deg^2^. For our stimuli, which were smoothly modulated by Gaussian envelopes, we approximate *a* with π · σ^2^, the area of a disk of the same radius as the standard deviation of the Gaussian envelope. *a*_*c*_ and *f*_0_ are the two parameters of the stimulus size model. We used the same equation but with different parameters for each color direction. We modeled the sensitivity using the OTF model from Rovamo et al. (1993, Equation 25) but found that it does not account for the drop in sensitivity at low frequencies and in our data.

Ideally, we would like to fit all five parameters of the model, but we found our data to be insufficient for that. Therefore, instead, we use the spatial integration parameters from the original article for achromatic sensitivity: *a*_0_ = 114 and *f*_0_ = 0.65. For the two chromatic sensitivities, we set *a*_0_ to 40 and *f*_0_ was kept the same as for the achromatic sensitivity. More data for large-size chromatic gratings would need to be collected to fully establish the values of these coefficients. As before, the data were fitted to the average observer data but only for chromatic frequencies up to 2 cpd. The model was fitted to the 20 cd/m^2^ data, which contained the variation in stimulus size (Experiment 4). The parameters of the model are presented in Table 5.

**Table 5.** Area-dependent parameters of log-parabola at 20 cd/m^2^.

	**Parameters**		
**Channel**	*S*_max_	*f*_max_	*b*
Achromatic	447.5	1.105	0.6764
Red-green	2780	0.1321	1.832
Yellow-violet	555.7	0.04399	2.397

**Figure 16.**
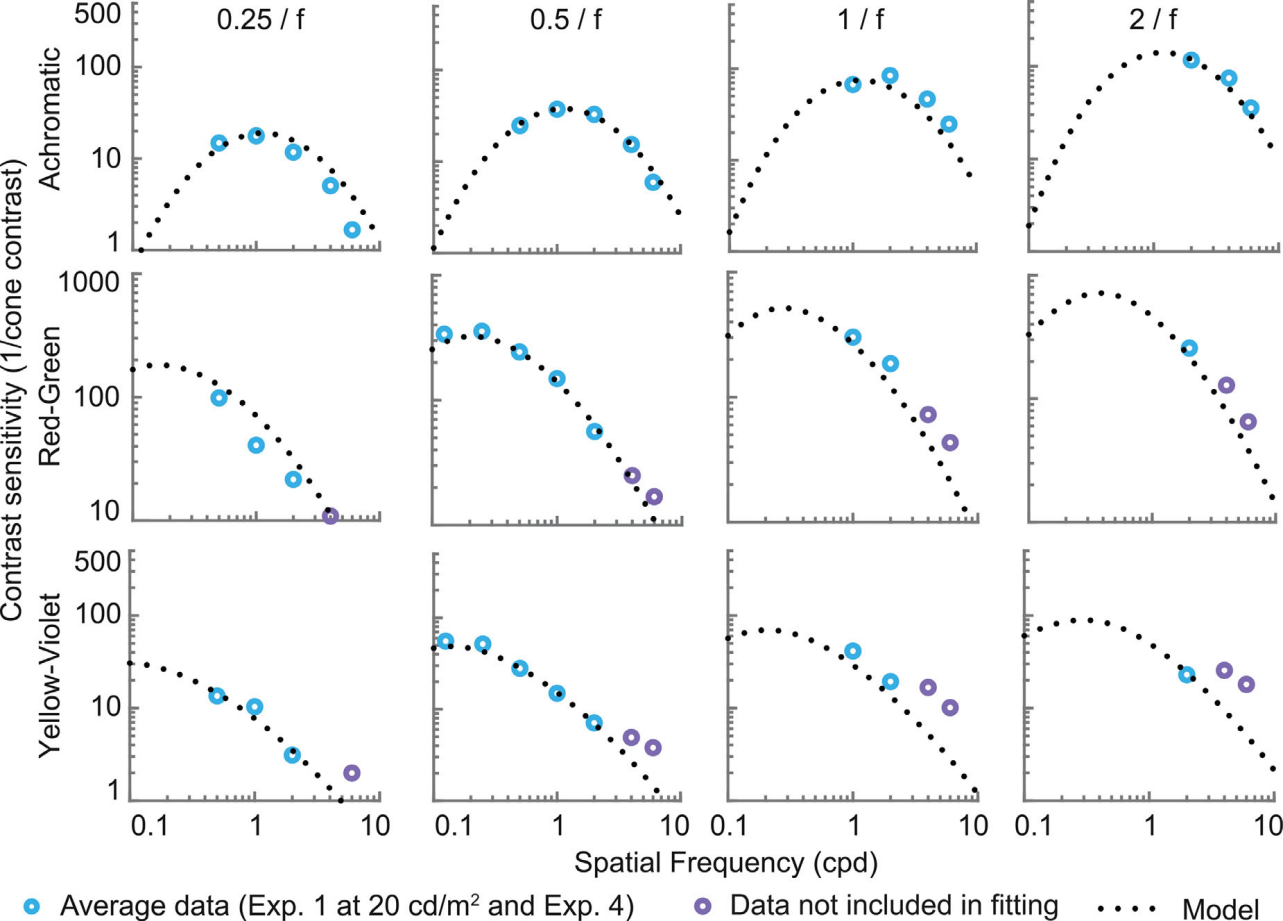
Contrast sensitivity predictions for fixed-cycles stimuli, compared to the results of Experiment 4. Each row represents a separate color direction. Each column is plotted for a different stimulus size, determined as a fraction of the wavelength. Higher frequency data points for chromatic channels are not included in the fitting.

**Figure 17.**
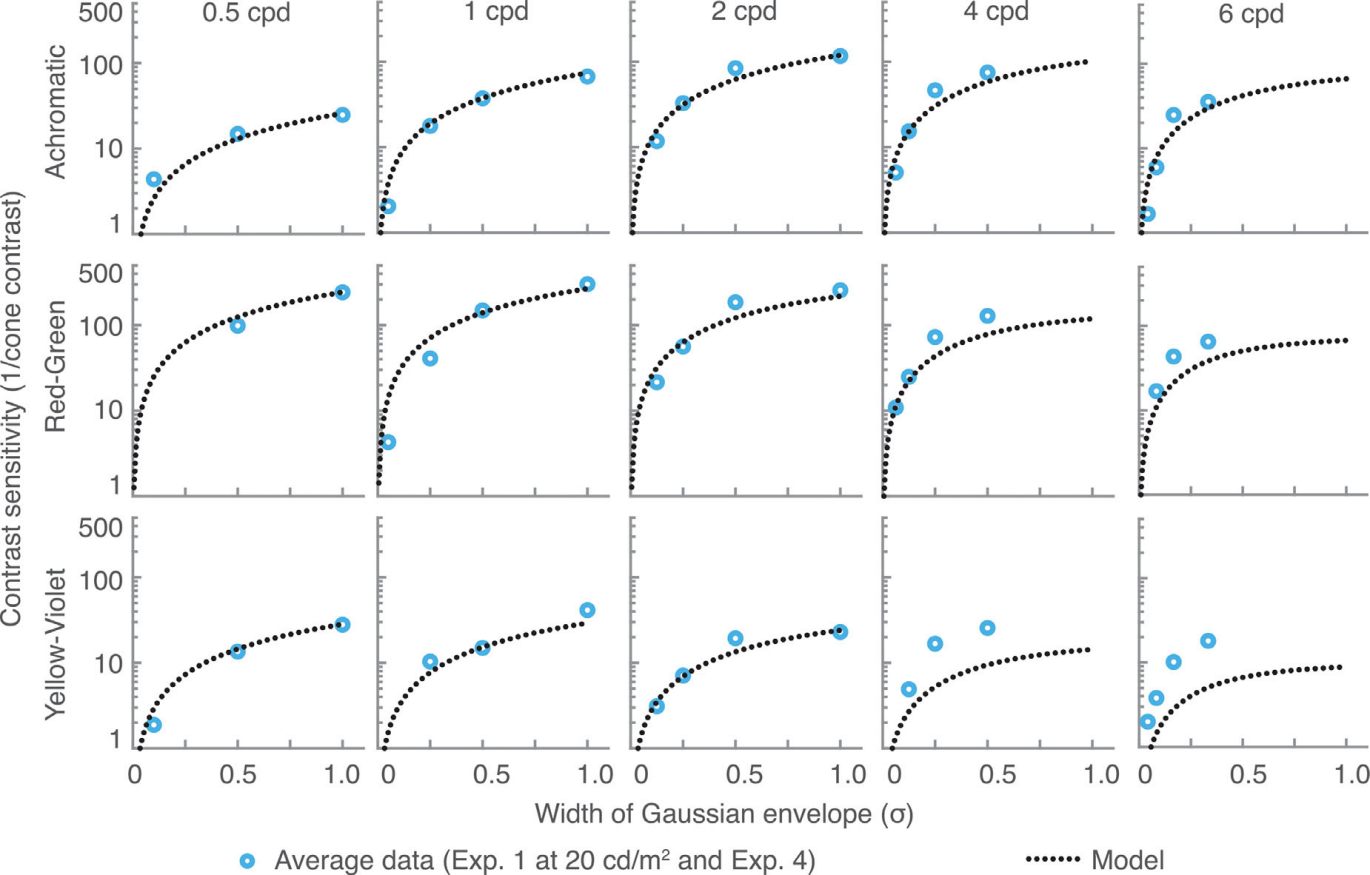
Contrast sensitivity predictions as a function of stimulus size (σ of the Gaussian envelope), compared with the results of Experiment 4. Each row shows predictions for a separate color direction. Each column is plotted for a different spatial frequency.

The fits to the data from Experiment 4 are shown in Figures 16 and 17. The model from Equation 17 accounts reasonably well for the size of both achromatic and chromatic stimuli. However, the predictions are less accurate at higher frequencies for the two chromatic channels. This is to be expected as we did not intend to fit these data points, which would require modeling luminance intrusion.

**Figure 18.**
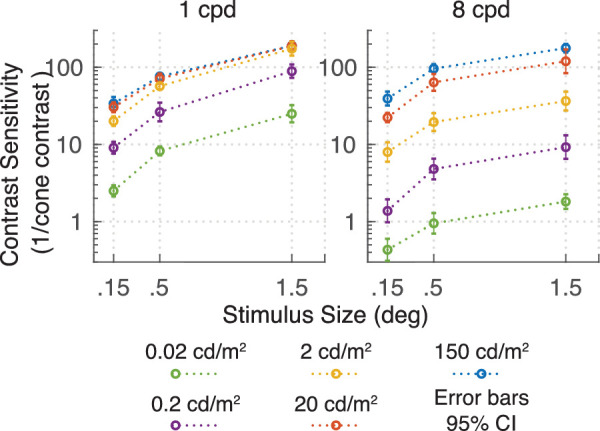
Achromatic contrast sensitivity at different luminance levels, as a function of stimulus size. From Mantiuk et al. (2011).

To use our model to predict data sets measured at different luminance levels, we extend the model to include the previously derived light-level dependency. Figure 18 shows the data from Mantiuk et al. (2011), where contrast sensitivity was measured at different luminance levels for stimuli of different extents. For a fixed spatial frequency, the sensitivity curve is simply shifted upward in log-log space, suggesting that there is little interaction between the effect of light level and the effect of stimulus size. Therefore, contrast sensitivity can be simply modeled:

(18) $$S_{AL}\left(f,l,a\right)=S_{A}\left(f,a\right)\cdot \frac{S_{L}\left(f,l\right)}{S_{L}\left(f,20\right)}$$

where *S*_*L*_ is luminance-dependent chromatic/achromatic CSF from the previous section (Equations 13–15) and *S*_*A*_ is the area-dependent CSF from Equation 17. The *S*_*L*_(*f*, 20) in the denominator accounts for the fact that *S*_*A*_ was fitted to the data measured at 20 cd/m^2^.

### 6.5 Comparison with other data sets

In the previous sections, we showed that a relatively simple model can predict contrast sensitivity variation due to frequency, stimulus size, and adapting luminance level, both for chromatic and achromatic gratings, as measured in our experiments. In this section, we demonstrate that the same model can generalize and predict data from other experiments. We selected data sets that contained variability in luminance levels and/or included both chromatic and achromatic stimuli.

**Figure 19.**
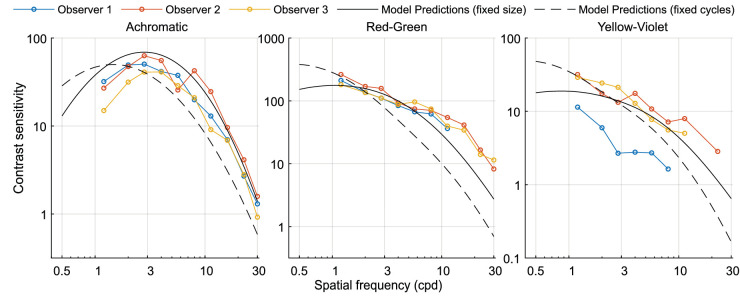
Comparison of our model with the ColorFest data set from Wuerger et al. (2002). The data are well explained by the continuous lines, showing the predictions for fixed-size stimuli, which were used in the original experiment.

First we use the model from Equation 18 to predict the data from the ColorFest study (Wuerger et al., 2002). It should be noted that the ColorFest study used stimuli of fixed size, and stimuli were temporally modulated (Gaussian modulation with a standard deviation of 0.125 s). The sensitivity in the ColorFest data is uniformly, across all three color directions, higher by a factor of 0.3 log_10_ units. To obtain comparable sensitivity values, we reduced the sensitivity of the original data by this amount, which resulted in reasonably good fits (Figure 19). The difference in overall sensitivity could be explained by the differences in experimental procedures: While ColorFest data were collected sequentially for each stimulus variation so that the same pattern was presented in consecutive 2AFC trials, in our 4AFC procedure, we randomly selected a stimulus of a different frequency, color direction, or orientation in each trial.

Figure 19 shows the original data together with the model predictions. Predictions for that data are shown as solid lines (labelled “fixed size”). In addition to that, we show as dashed lines the predictions for the stimuli with the fixed number of cycles (and varying size), similar to the stimuli used in our experiments (labeled “fixed cycles”). The model from Equation 18 was used for both curves.

**Figure 20.**
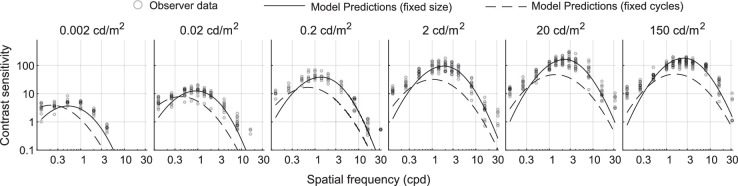
Comparison of our model predictions with the achromatic contrast sensitivity measurements from Mantiuk et al. (2011). Solid lines represent the same stimuli as used for the measurements.

**Figure 21.**
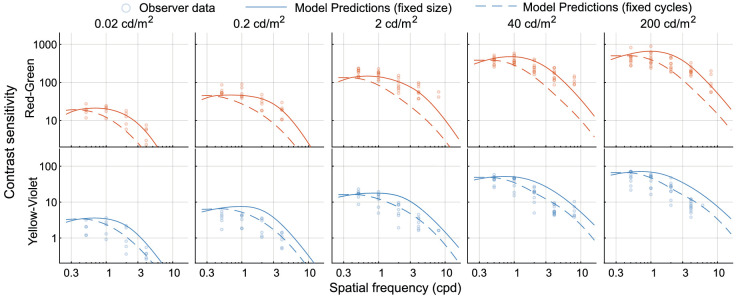
Comparison of our model predictions with chromatic contrast sensitivity measurements from Kim et al. (2013). Solid lines represent the same stimuli as used for the measurements.

Finally, we use the model to predict the data from the measurements of achromatic and chromatic gratings at luminance levels varying from 0.002 cd/m^2^ to 200 cd/m^2^ from Kim et al. (2013). Since the experimental procedure was the same as in Wuerger et al. (2002) and different from the experiments reported in the current article, we reduced the contrast sensitivity of the data by the same amount of 0.3 log_10_ units. The predictions for achromatic gratings are shown in Figure 20 and for chromatic gratings in Figure 21. We use the same notation as before: solid lines for fixed-size stimuli used in Kim et al. (2013) experiments and dashed line for the fixed-cycles stimuli used in our experiment. The predictions of the model (solid lines) for achromatic gratings are close to the data except for the two lowest frequencies. This could be both due to the limitation of the simple log-parabola model we use and the lack of data for low frequencies and achromatic gratings. The predictions for chromatic gratings (Figure 21) are reasonably accurate for the red-green color direction but slightly higher than the measurements for the yellow-violet color direction. We could not determine the cause of that difference.

## Discussion

Spatial contrast sensitivity is one of the most basic measures of visual performance: It determines the minimum contrast required for observers to detect spatial patterns at different spatial scales. Spatial CSFs have applications in clinical settings as well as in optimizing display technologies based on the known limitations of the human visual system. For that reason, CSFs have been studied extensively since the seminal article by Campbell and Robson (1968). Most of these studies have focused on contrast sensitivity at modest photopic light levels (usually ranging from about 10 to 50  cd/m^2^) and a comprehensive model for achromatic spatial detection mechanisms has been proposed (Watson & Ahumada, 2005).

In the natural environment, our visual system needs to operate over a large dynamic range, from starlight to bright sunlight. This is achieved by light adaptation within the retina, which ensures a useful dynamic range in the cone photoreceptor system (for a review, see Barbur & Stockman, 2010). Van Nes and Bouman (1967) measured spatial contrast sensitivity over a wide range of retinal illuminances (from 0.0009 to 5,900 trolands) and observed that contrast sensitivity increases steadily with ambient illumination, up to about 900 trolands, where the sensitivity seems to saturate, reflecting light adaptation in the cone receptors. Second, contrast sensitivity for low spatial frequencies saturates earlier (at around 0.09 trolands) than for higher spatial frequencies, probably reflecting a decrease in spatial integration with increasing light level.

**Figure 22.**
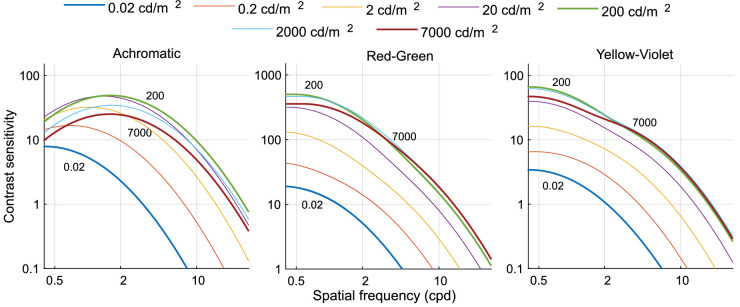
Summary of our model for spatio-chromatic contrast sensitivity at multiple luminance levels.

Broadly speaking, our results from Experiment 1 are consistent with Van Nes and Bouman (1967) but extend these findings in two important aspects. First, we measured the CSFs not only for achromatic stimulus modulations but also for chromatic variations (red-green, yellow-violet). Second, since we were able to measure the CSFs at higher light levels than was previously possible (0.86 to 36,000 trolands reflecting outdoor light levels), we could probe at which retinal illuminance the CSF saturates. We find the same pattern of results, that is, achromatic contrast sensitivity is steadily increasing with increasing light level (Figure 22). However, in contrast to the findings by Van Nes and Bouman (1967), for comparable spatial frequencies, the sensitivity seems to reach its peak somewhere between 2,000 and 3,000 trolands and then decreases at even higher illumination levels (cf. Figure 7), consistent with recent findings by Bierings et al. (2019). For chromatic stimulus modulations, the contrast sensitivity seems to reach its peak at about 2,000 trolands and then saturates, broadly consistent with a Weber-law behavior, and previous measurements using interference fringes (Sekiguchi et al., 1993). There is some suggestion in the chromatic data that contrast thresholds are also increasing with increasing light levels, but the inflection point is at higher light levels than for the achromatic data (cf. Figure 7).

We can only speculate on the cause of Weber law failure at high photopic light levels and whether this decrease in sensitivity is related to bleaching or pigment depletion. Experiment 2 was designed to test whether incomplete adaptation could play a role, but our data do not support this explanation (Figure 8). The larger sensitivity loss in the achromatic compared to the chromatic pathways at high retinal illuminance levels is consistent with the idea that a sensitivity loss at the cone level has a more pronounced effect on the achromatic pathway (due to summing L- and M-cone outputs) compared to the chromatic pathways where differences of cone outputs are computed.

### Further developments of the contrast sensitivity model

Most of our measurements (Experiment 1) were based on fixed-cycles as opposed to fixed-size stimuli, the former being preferable since fixed-cycles stimuli are more likely to reflect the summation behavior of the bandpass spatial-frequency channels in the human visual system. To predict contrast sensitivity for stimuli of arbitrary size, we collected additional data with stimuli of different extents at one particular luminance level (20 cd/m^2^; Experiment 4). Adapting the model by Rovamo et al. (1993) allowed us to fit the size-varying data for both the achromatic and chromatic modulations but also to empirically test the size-dependent model by predicting previously collected data sets (Figure 19). To generalize the size-dependent model to arbitrary illumination levels, we made use of existing size-dependent contrast sensitivity measurements obtained at low mesopic and photopic light levels (Figure 18). For this luminance range (0.02 to 150  cd/m^2^) and size range (0.15 to 1.5 deg), the effect of size on contrast sensitivity is independent of the luminance level and can be modeled by a vertical shift in log-log space. The extended CSF model was tested by predicting achromatic CS data (Figure 20; Mantiuk et al., 2011) and chromatic data (Figure 21; Kim et al., 2013). Low and behold, the predictions are acceptable in particular when considering the different experimental methods and observer sample. Achromatic and red-green Contrast Sensitivity (CS) data are always better predicted by the size-dependent model, whereas the fixed-cycles predictions are slightly superior for the yellow-violet CS data. We have currently no solid explanation for this difference, but it may be due to possible light-level dependent differences in spatial integration mechanisms for red-green and yellow-violet modulations.

Finally, a model applicable to arbitrary spatio-chromatic images or natural scenes will also need to characterize the summation across the chromatic and luminance channels at detection threshold and how summation is modulated by retinal illuminance and stimulus size. While we have measured the CS for achromatic and chromatic stimuli in isolation, we have allowed for luminance intrusion in the detection of the nominally isoluminant chromatic contrast variations. The role of luminance artifacts in the detection of the nominally isoluminant chromatic stimuli is most apparent in the S-cone insolating gratings at medium to high luminance levels for frequencies beyond 2 cpd (Figure 13). We have modeled this interaction by assuming probability summation between the luminance and chromatic channel (Equation 7). Summation across luminance and chromatic channels and between chromatic channels needs to be further investigated by using more diagnostic contrast variations, that is, stimulus variations that are modulated in intermediate directions in threshold space.

### Low-pass shape of the chromatic contrast sensitivity function

Experiment 3 was designed to further probe the low-pass shape of the chromatic CSF by measuring thresholds at additional low frequencies (0.125, 0.25 cpd) for the very low mesopic (0.02  cd/m^2^) and high photopic illumination levels (7,000  cd/m^2^). We find no convincing evidence for a drop in sensitivity at the lowest frequency, hence confirming the low-pass shape of the chromatic CSF, consistent with Mullen (1985).

CS is a measure of performance at threshold. Models relating detection thresholds to suprathreshold appearance have been proposed with limited success, most notably the perceived-contrast model by Kulikowski (1976), which assumes that perceived contrast is related linearly to physical contrast once detection threshold has been subtracted. More recently, Shapley et al. (2019) have argued that, for chromatic stimuli, detection and suprathreshold appearance are mediated by different mechanisms drawing on distinct neuronal populations (single-opponent nonoriented vs. double-opponent orientation-tuned neurons): Contrast sensitivity at threshold is likely to be mediated by single-opponent neurons with a spatially low-pass characteristic, whereas suprathreshold appearance draws on double-opponent neurons that are sensitive to edges. If it is indeed the case that suprathreshold chromatic mechanisms do not exhibit the same low-pass shape as seen in the chromatic CSF, spatio-chromatic appearance models predicting perceptual attributes such as perceived contrast, colorfulness, and sharpness based on detection performance are unlikely to succeed. Double-opponent neurons encode medium spatial frequencies for both achromatic and isoluminant red-green stimuli and may be the neural substrate for the commensurate performance and contrast dependence for orientation discrimination (Wuerger & Morgan, 1999) and blur discrimination (Wuerger et al., 2001) for suprathreshold achromatic and red-green gratings.

### What the eyes see best

The motive in asking what stimulus the eyes see best is that it reveals the spatio-chromatic receptive field structure of the visual neurons that detect that stimulus. Watson et al. (1983) searched a large parameter space and concluded that, for achromatic sinusoidal modulations presented on a high-luminance background (340  cd/m^2^), the optimal spatial frequency was at 6 cpd and could be detected at a threshold contrast of 1.44%. Chaparro et al. (1993) generalized their study by including chromatic and achromatic stimuli of various stimulus sizes and durations, presented on a bright yellow background (3,000 trolands). The optimal duration and stimulus size were greater for the chromatic spots compared to the achromatic ones, consistent with greater temporal and spatial summation. However, even for the nonoptimal parameter settings, the threshold contrasts for chromatic variations were consistently lower (by a factor of 5–9) than for achromatic spots. The lowest threshold contrast (defined as cone contrast; see Equation 1) was 0.7% for chromatic stimuli and 3% for achromatic variations. Our measurements (cf. Figure 7) confirm the superior sensitivity to chromatic contrast variations. The lowest threshold contrast (0.2% cone contrast) is reached at 2,000 trolands for a low spatial frequency (0.5 cpd) chromatic stimulus; for achromatic variations, the best detection performance (lowest threshold: 2%) is also achieved at 2,000 trolands but at a medium spatial frequency (2 cpd). The superior sensitivity to chromatic over achromatic variations (by a factor of 10 in our experiment) is consistent with the prevalence of retinal parvocellular neurons, which are L/M cone-opponent. It is worth noting that the cone contrast measure used to compare chromatic and achromatic variations does not reflect the contrast variations found in natural scenes (Burton & Moorhead, 1987; Párraga et al., 1998); the high chromatic sensitivity of the visual system might rather compensate for the low chromatic contrasts typically occurring in our natural environment (Chaparro et al., 1993).

## Summary and conclusions

Spatial contrast sensitivity measurements are commonly used to characterize the sensitivity of the human visual system at different spatial scales. We have extended existing measurements of contrast sensitivity to cover light levels ranging from low mesopic (0.02 cd/m^2^) to high photopic (7,000 cd/m^2^) levels and, crucially, measured sensitivity as a function of light level in all three directions of color space, an achromatic direction and two chromatic ones (red-green, yellow-violet).

All our measurements were performed under steady-state adaptation to a particular light level. A notable feature of these extended contrast sensitivity measurements is that the adapting light level has a differential effect on the chromatic and achromatic contrast sensitivity in several important aspects: (a) We extended the contrast sensitivity measurements by Van Nes et al. (1967) and demonstrated that the achromatic contrast sensitivity does not saturate at 200 cd/m^2^, but it decreases again at higher light levels (Figure 22). (b) The light level at which Weber-law behavior was observed was frequency dependent for achromatic stimuli (2 cd/m^2^ for 0.5 cpd; 200 cd/m^2^ for 6 cpd), whereas for chromatic sensitivity, we observed the transition to Weber law to occur at about 200 cd/m^2^ at all spatial frequencies (Figure 7). (c) We extended the chromatic contrast sensitivity measurements of Mullen (1985) to very low and high light levels and showed that chromatic sensitivity saturates at about 200 cd/m^2^ for spatial frequencies above 1 cpd.

We used these contrast sensitivity measurements, in conjunction with supplementary measurements on spatial summation in both the chromatic and achromatic domain, to derive a computational CSF model that predicts spatial contrast sensitivity for ambient light levels ranging from low mesopic and to high photopic levels. Our CSF model reflects the visual system of an average (standard) observer, hence affording the generality necessary for practical applications in display technology as well as providing comparative data for clinical investigations.

## Footnotes

1 Tabulated cone fundamentals can be found at http://cvrl.ucl.ac.uk/.

## References

[bib1] Ahumada A. J. Jr. Peterson H. A. (1992). Luminance-model-based DCT quantization for color image compression. In Rogowitz B. E. (Ed.), *Human vision, visual processing, and digital display III* (Vol. 1666, pp. 365–374). International Society for Optics and Photonics, San Jose, CA, USA.

[bib2] Anderson S. J., Mullen K. T., & Hess R. F. (1991). Human peripheral spatial resolution for achromatic and chromatic stimuli: limits imposed by optical and retinal factors. *Journal of Physiology,* 442, 47–64. 1798037 10.1113/jphysiol.1991.sp018781 PMC1179877

[bib3] Andrews B. W., & Pollen D. A. (1979). Relationship between spatial-frequency selectivity and receptive-field profile of simple cells. *Journal of Physiology,* 287, 163–176. 430391 10.1113/jphysiol.1979.sp012652 PMC1281488

[bib4] Barbur J., & Stockman A. (2010). Photopic, mesopic and scotopic vision and changes in visual performance. In Dartt D. A. (Ed.), *Encyclopedia of the eye,* (pp. 323–331). Oxford, UK: Academic Press.

[bib5] Berns R. S. (1996). Methods for characterizing CRT displays. *Displays,* 16, 173–182.

[bib6] Bierings R., Overkempe T., Berkel C., Kuiper M., & Jansonius N. (2019). Spatial contrast sensitivity from star-to sunlight in healthy subjects and patients with glaucoma. *Vision Research,* 158, 31–39. 30721742 10.1016/j.visres.2019.01.011

[bib7] Bilodeau L., & Faubert J. (1997). Isoluminance and chromatic motion perception throughout the visual field. *Vision Research,* 37, 2073–2081. 9327055 10.1016/s0042-6989(97)00012-6

[bib8] Brainard D. H. (1996). Cone contrast and opponent modulation color spaces. *Human Color Vision*. Kaiser P. K., Boynton R. M. (Eds.), 2nd edn. Optical Society of America: Washington, DC, Part IV, pp. 563–579.

[bib9] Burton G. J., & Moorhead I. R. (1987). Color and spatial structure in natural scenes. *Applied Optics,* 26, 157–170. 20454092 10.1364/AO.26.000157

[bib10] Campbell F. W., Kulikowski J. J., & Levinson J. (1966). The effect of orientation on the visual resolution of gratings. *Journal of Physiology,* 187, 427–436. 5972182 10.1113/jphysiol.1966.sp008100 PMC1395930

[bib11] Campbell F. W., & Robson J. (1968). Application of fourier analysis to the visibility of gratings. *Journal of Physiology,* 197, 551. 5666169 10.1113/jphysiol.1968.sp008574 PMC1351748

[bib12] Capilla P., Malo J., Luque M. J., & Artigas J. M. (1998). Colour representation spaces at different physiological levels: a comparative analysis. *Journal of Optics,* 29, 324–338.

[bib13] Chaparro A., Stromeyer C., Huang E., Kronauer R., & Eskew R. (1993). Colour is what the eye sees best. *Nature,* 361, 348–350. 8426653 10.1038/361348a0

[bib14] CIE. (2006). *Fundamental chromacity diagram with psychological axes—part 1*. Technical report, Central Bureau of the Commission Internationale de l’ Éclairage.

[bib15] Cole G. R., Hine T., & McIlhagga W. (1993). Detection mechanisms in L-, M-, and S-cone contrast space. *Journal of the Optical Society of America A,* 10, 38–51. 10.1364/josaa.10.000038 8478744

[bib16] Cropper S. J. (1998). Detection of chromatic and luminance contrast modulation by the visual system. *Journal of the Optical Society of America A,* 15, 1969–1986. 10.1364/josaa.15.001969 9691482

[bib17] De Vries H. (1943). The quantum character of light and its bearing upon threshold of vision, differential sensitivity and visual acuity of the eye. *Physica,* 10, 553–564.

[bib18] Derrington A. M., Krauskopf J., & Lennie P. (1984). Chromatic mechanisms in lateral geniculate nucleus of macaque. *Journal of Physiology,* 357, 241–265. 6512691 10.1113/jphysiol.1984.sp015499 PMC1193257

[bib19] Díez-Ajenjo M. A., & Capilla P. (2010). Spatio-temporal contrast sensitivity in the cardinal directions of the colour space: A review. *Journal of Optometry,* 3, 2–19.

[bib20] Flitcroft D. I. (1989). The interactions between chromatic aberration, defocus and stimulus chromaticity: Implications for visual physiology and colorimetry. *Vision Research,* 29, 349–360. 2773345 10.1016/0042-6989(89)90083-7

[bib21] Gibson K. S., & Tyndall E. P. T. (1923). Visibility of radiant energy. *Scientific Papers of the Bureau of Standards,* 19, 131–191.

[bib22] Graham C. H., & Margaria R. (1935). Area and the intensity-time relation in the peripheral retina. *American Journal of Physiology—Legacy Content,* 113, 299–305.

[bib23] Granger E. M., & Heurtley J. C. (1973). Visual chromaticity-modulation transfer function. *Journal of the Optical Society of America,* 63, 1173–1174. 4748547 10.1364/josa.63.001173

[bib24] Green D. G. (1968). The contrast sensitivity of the colour mechanisms of the human eye. *Journal of Physiology,* 196, 415–429. 5652884 10.1113/jphysiol.1968.sp008515 PMC1351720

[bib25] Hoekstra J., van der Goot D., van den Brink G., & Bilsen F. (1974). The influence of the number of cycles upon the visual contrast threshold for spatial sine wave patterns. *Vision Research,* 14, 365–368. 4854900 10.1016/0042-6989(74)90234-x

[bib26] Howell E., & Hess R. (1978). The functional area for summation to threshold for sinusoidal gratings. *Vision Research,* 18, 369–374. 664315 10.1016/0042-6989(78)90045-7

[bib27] Ikeda M., & Shimozono H. (1981). Mesopic luminous-efficiency functions. *Journal of the Optical Society of America,* 71, 280–284. 7218072 10.1364/josa.71.000280

[bib28] Kim K. J., Mantiuk R., & Lee K. H. (2013). Measurements of achromatic and chromatic contrast sensitivity functions for an extended range of adaptation luminance. In Rogowitz B. E. Pappas T. N. de Ridder H. (Eds.), *Human vision and electronic imaging XVIII* (Vol. 8651, pp. 319–332). International Society for Optics and Photonics, SPIE Burlingame, California, USA.

[bib29] Kim Y. J., Reynaud A., Hess R. F., & Mullen K. T. (2017). A normative data set for the clinical assessment of achromatic and chromatic contrast sensitivity using a qCSF approach. *Investigative Ophthalmology & Visual Science,* 58, 3628–3636. 28728170 10.1167/iovs.17-21645

[bib30] Kleiner M., Brainard D., Pelli D., Ingling A., Murray R., & Broussard C. (2007). What's new in psychtoolbox-3?. *Perception,* 36(14), 1–16.

[bib31] Kulikowski J. J. (1976). Effective contrast constancy and linearity of contrast sensation. *Vision Research,* 16, 1419–1431. 1007022 10.1016/0042-6989(76)90161-9

[bib32] Lucassen M., Lambooij M., Sekulovski D., & Vogels I. (2018). Spatio-chromatic sensitivity explained by post-receptoral contrast. *Journal of Vision,* 18, 13–13, 10.1167/18.5.13. 29904788

[bib33] Luntinen O., Rovamo J., & Näsänen R. (1995). Modelling the increase of contrast sensitivity with grating area and exposure time. *Vision Research,* 35, 2339–2346. 7571469 10.1016/0042-6989(94)00309-a

[bib34] Manahilov V., Simpson W. A., & McCulloch D. L. (2001). Spatial summation of peripheral gabor patches. *Journal of the Optical Society of America A,* 18, 273–282. 10.1364/josaa.18.000273 11205972

[bib35] Mantiuk R., Kim K. J., Rempel A. G., & Heidrich W. (2011). HDR-VDP-2: A calibrated visual metric for visibility and quality predictions in all luminance conditions. *ACM Transactions on Graphics,* 30, 40, doi:10.1145/2010324.1964935.

[bib36] McKeefry D. J., Murray I. J., & Kulikowski J. J. (2001). Red-green and blue-yellow mechanisms are matched in sensitivity for temporal and spatial modulation. *Vision Research,* 41, 245–255. 11163858 10.1016/s0042-6989(00)00247-9

[bib37] Meese T. S., & Summers R. J. (2007). Area summation in human vision at and above detection threshold. *Proceedings of the Royal Society B: Biological Sciences,* 274, 2891–2900. 10.1098/rspb.2007.0957 PMC2211515 17851151

[bib38] Mollon J. D., & Reffin J. (1989). A computer-controlled color-vision test that combines the principles of Chibret and of Stilling. *Journal of Physiology—London,* 414, 5.

[bib39] Mullen K. (1985). The contrast sensitivity of human colour vision to red-green and blue-yellow chromatic gratings. *Journal of Physiology,* 359, 381–440. 3999044 10.1113/jphysiol.1985.sp015591 PMC1193381

[bib40] Mullen K. (1991). Colour vision as a post-receptoral specialization of the central visual field. *Vision Research,* 31, 119–130. 2006545 10.1016/0042-6989(91)90079-k

[bib41] Mustonen J., Rovamo J., & Näsänen R. (1993). The effects of grating area and spatial frequency on contrast sensitivity as a function of light level. *Vision Research,* 33, 2065–2072. 8266648 10.1016/0042-6989(93)90005-h

[bib42] Noorlander C., Heuts M. G., & Koenderink J. J. (1980). Influence of the target size on the detection threshold for luminance and chromaticity contrast. *Journal of the Optical Society of America*, 1116–1121, 10.1364/JOSA.70.001116. 7411269

[bib42a] Párraga C. A., Brelstaff G., Troscianko T., & Moorehead I. R. (1998). Color and luminance information in natural scenes. *Journal of the Optical Society of America A,* 15(3), 563–569, 10.1364/JOSAA.15.000563. 9499586

[bib43] Piper H. (1903). Über die Abhängigkeit des Reizwertes leuchtender Objekte von ihrer Flächen-bezw. Winkelgröße. [The effect of area and visual angle on the sensation of self-luminous objects] *Zeitschrift fur Psychologie und Physiologie der Sinnesorgane,* 32, 98–122.

[bib44] Robson J. G., & Graham N. V. S. (1981). Probability summation and regional variation in contrast sensitivity across the visual field. *Vision Research,* 21, 409–418. 7269319 10.1016/0042-6989(81)90169-3

[bib45] Rohaly A. M., & Owsley C. (1993). Modeling the contrast-sensitivity functions of older adults. *Journal of the Optical Society of America A,* 10, 1591–1599. 10.1364/josaa.10.001591 8350148

[bib46] Rose A. (1948). The sensitivity performance of the human eye on an absolute scale. *Journal of the Optical Society of America,* 38, 196–208. 18901781 10.1364/josa.38.000196

[bib47] Rovamo J., Luntinen O., & Näsänen R. (1993). Modelling the dependence of contrast sensitivity on grating area and spatial frequency. *Vision Research,* 33, 2773–2788. 8296472 10.1016/0042-6989(93)90235-o

[bib48] Seetzen H., Heidrich W., Stuerzlinger W., Ward G., Whitehead L., Trentacoste M., Ghosh A., & Vorozcovs A. (2004). High dynamic range display systems. *ACM Transactions on Graphics,* 23, 760.

[bib49] Sekiguchi N., Williams D. R., & Brainard D. H. (1993). Efficiency in detection of isoluminant and isochromatic interference fringes. *Journal of the Optical Society of America A,* 10, 2118. 10.1364/josaa.10.002118 8229351

[bib50] Shapley R., & Hawken M. J. (2011). Color in the cortex: Single- and double-opponent cells. *Vision Research,* 51, 701–717. 21333672 10.1016/j.visres.2011.02.012 PMC3121536

[bib51] Shapley R., Nunez V., & Gordon J. (2019). Cortical double-opponent cells and human color perception. *Current Opinion in Behavioral Sciences,* 30, 1–7.

[bib52] Shlaer S. (1937). The relation between visual acuity and illumination. *Journal of General Physiology,* 21, 165–188. 19873045 10.1085/jgp.21.2.165 PMC2141937

[bib53] Swanson W. H. (1996). S-cone spatial contrast sensitivity can be independent of pre-receptoral factors. *Vision Research,* 36, 3549–3555. 8977021 10.1016/0042-6989(96)00047-8

[bib54] To M. P. S., & Tolhurst D. J. (2019). V1-based modeling of discrimination between natural scenes within the luminance and isoluminant color planes. *Journal of Vision,* 19(1), 9, 10.1167/19.1.9 30650432

[bib55] Valero E. M., Nieves J. L., Hernndez-Andrs J., & Garca J. A. (2004). Changes in contrast thresholds with mean luminance for chromatic and luminance gratings: A reexamination of the transition from the devriesrose to weber regions. *Color Research & Application,* 29, 177–182.

[bib56] van der Horst G. J. C., & Bouman M. A. (1969). Spatiotemporal chromaticity discrimination. *Journal of the Optical Society of America,* 59, 1482–1488. 5357930 10.1364/josa.59.001482

[bib57] Van Nes F. L., & Bouman M. A. (1967). Spatial modulation transfer in the human eye. *Journal of the Optical Society of America,* 57, 401–406. 10.1364/josa.57.001082 6051762

[bib58] Van Nes F. L., Koenderink J. J., Nas H., & Bouman M. A. (1967). Spatiotemporal modulation transfer in the human eye. *Journal of the Optical Society of America,* 57, 1082. 6051762 10.1364/josa.57.001082

[bib59] Vangorp P., Myszkowski K., Graf E. W., & Mantiuk R. K. (2015). A model of local adaptation. *ACM Transactions on Graphics,* 34, 1–13.

[bib60] Vassilev A., Zlatkova M., Manahilov V., Krumov A., & Schaumberger M. (2000). Spatial summation of blue-on-yellow light increments and decrements in human vision. *Vision Research,* 40, 989–1000. 10720668 10.1016/s0042-6989(99)00220-5

[bib61] Wagner G., & Boynton R. M. (1972). Comparison of four methods of heterochromatic photometry. *Journal of the Optical Society of America,* 62, 1508–1515. 4643012 10.1364/josa.62.001508

[bib62] Watson A. B., & Ahumada A. J. (2005). A standard model for foveal detection of spatial contrast. *Journal of Vision,* 5, 717–740, 10.1167/5.9.6. 16356081

[bib63] Watson A. B., Barlow H., & Robson J. (1983). What does the eye see best? *Nature,* 302, 419–422. 6835375 10.1038/302419a0

[bib64] Watson A. B., & Pelli D. G. (1983). Quest: A Bayesian adaptive psychometric method. *Perception & Psychophysics,* 33, 113–120. 6844102 10.3758/bf03202828

[bib65] Watson A. B., & Yellott J. I. (2012). A unified formula for light-adapted pupil size. *Journal of Vision,* 12, 12–12, 10.1167/12.10.12. 23012448

[bib66] Wuerger S., & Morgan M. (1999). Input of long- and middle-wavelength-sensitive cones to orientation discrimination. *Journal of the Optical Society of America A,* 16, 436–442.

[bib67] Wuerger S., Owens H., & Westland S. (2001). Blur tolerance for luminance and chromatic stimuli. *Journal of the Optical Society of America A,* 18, 1231–1239. 10.1364/josaa.18.001231 11393614

[bib68] Wuerger S., Watson A., & Ahumada A. (2002). Towards a spatio-chromatic standard observer for detection. In *Proceedings of SPIE—The International Society for Optical Engineering* (Vol. 4662).

[bib68a] Wuerger S. M., Watson A. B., Ahumada A. J. Jr. (2002). Towards a spatio-chromatic standard observer for detection. *Proc. SPIE 4662, Human Vision and Electronic Imaging VII, (30 May 2002)*, Rogowitz B. E., Pappas T. N. (Eds.), San Jose, California, USA, 10.1117/12.469512.

